# Analysis of gene expression and mutation data points on contribution of transcription to the mutagenesis by APOBEC enzymes

**DOI:** 10.1093/narcan/zcab025

**Published:** 2021-07-02

**Authors:** Almira Chervova, Bulat Fatykhov, Alexander Koblov, Evgeny Shvarov, Julia Preobrazhenskaya, Dmitry Vinogradov, Gennady V Ponomarev, Mikhail S Gelfand, Marat D Kazanov

**Affiliations:** Institute of Oncology, Radiology and Nuclear Medicine, Dmitry Rogachev National Medical Research Center of Pediatric Hematology, Oncology and Immunology, Moscow, 117997, Russia; Department of Developmental and Stem Cell Biology, Institut Pasteur, Paris, 75015, France; Department of Control and Applied Mathematics, Moscow Institute of Physics and Technology, Dolgoprudny, 141700, Russia; InterSystems Corporation, Cambridge MA, 02142, USA; InterSystems Corporation, Cambridge MA, 02142, USA; Faculty of Bioengineering and Bioinformatics, Lomonosov Moscow State University, Moscow, 119234, Russia; Research and Training Center of Bioinformatics, Institute for Information Transmission Problems (the Kharkevich Institute, RAS), Moscow, 127051, Russia; Research and Training Center of Bioinformatics, Institute for Information Transmission Problems (the Kharkevich Institute, RAS), Moscow, 127051, Russia; Research and Training Center of Bioinformatics, Institute for Information Transmission Problems (the Kharkevich Institute, RAS), Moscow, 127051, Russia; Center of Life Sciences, Skolkovo Institute of Science and Technology, Moscow, 121205, Russia; Research and Training Center of Bioinformatics, Institute for Information Transmission Problems (the Kharkevich Institute, RAS), Moscow, 127051, Russia; Center of Life Sciences, Skolkovo Institute of Science and Technology, Moscow, 121205, Russia

## Abstract

Since the discovery of the role of the APOBEC enzymes in human cancers, the mechanisms of this type of mutagenesis remain little understood. Theoretically, targeting of single-stranded DNA by the APOBEC enzymes could occur during cellular processes leading to the unwinding of DNA double-stranded structure. Some evidence points to the importance of replication in the APOBEC mutagenesis, while the role of transcription is still underexplored. Here, we analyzed gene expression and whole genome sequencing data from five types of human cancers with substantial APOBEC activity to estimate the involvement of transcription in the APOBEC mutagenesis and compare its impact with that of replication. Using the TCN motif as the mutation signature of the APOBEC enzymes, we observed a correlation of active APOBEC mutagenesis with gene expression, confirmed the increase of APOBEC-induced mutations in early-replicating regions and estimated the relative impact of transcription and replication on the APOBEC mutagenesis. We also found that the known effect of higher density of APOBEC-induced mutations on the lagging strand was highest in middle-replicating regions and observed higher APOBEC mutation density on the sense strand, the latter bias positively correlated with the gene expression level.

## INTRODUCTION

Apolipoprotein B mRNA editing catalytic polypeptide-like (APOBEC) is a family of enzymes of the human innate immune system, whose known role is the defense against viruses and transposable elements (1). The APOBEC enzymes bind to single-stranded viral DNA and deaminate cytosine, leading to C > T and C > G substitutions in the TpC context (2). Recently, APOBEC enzymes have been implicated in cancer mutagenesis (3–5) with APOBEC-associated mutations detected in many types of human cancer, including breast, lung, bladder, head/neck and cervical cancers (6–8). In a majority of these cancer genomes, the APOBEC-signature mutations were found clustered in DNA and located on the same DNA strand (3,4). In addition, cancer genomes enriched in APOBEC-induced mutations also contain mutations with the APOBEC signature that are not positionally clustered along the genome. As the APOBEC enzymes have a strong specificity toward single-stranded DNA (ssDNA), it has been suggested that the enzymes generate mutation during one or several cellular processes associated with the unwinding of double-stranded human DNA, such as DNA repair, replication or transcription (9). However, the exact mechanisms of APOBEC-associated mutagenesis remain unknown (10).

During DNA replication, ssDNA regions are transiently formed behind the replication fork and theoretically can serve as a substrate for the APOBEC enzymes. Furthermore, nucleotide polymerization on the lagging strand runs in the opposite direction and requires formation of ssDNA loops (11,12). Indeed, recent papers indicate that the APOBEC mutagenesis is associated with replication, as the density of APOBEC-induced mutations has a strong bias toward the lagging replication strand (13) and is relatively higher in early-replicated regions (14).

During transcription, RNA polymerase binds to the antisense strand of DNA, leaving the other, sense strand single-stranded and hence potentially exposed to the APOBEC mutagenesis. Additionally, formation of R-loops, triple-stranded nucleic acid structures comprised synthetized RNA hybridized with the DNA antisense, and single-stranded sense DNA (15), may facilitate the APOBEC access to the transient ssDNA in the non-transcribed strand. The first evidence for the link between the APOBEC mutagenesis and transcription was obtained in whole-genome, exome and transcriptome study of bladder cancer (16) that demonstrated the correlation of APOBEC-signature mutation rate with the mean expression level, and the bias toward the sense strand. Recent study in yeasts demonstrated susceptibility of the sense strand of tRNA genes to APOBEC mutagenesis, which were mutated 1000-fold times more frequently than the non-tRNA genomic regions (17). On the other hand, a study analyzing the distribution of APOBEC-induced mutations across genomes of 119 breast and 24 lung cancer samples (14) did not find statistically significant difference of the density of APOBEC-induced mutations between transcribed and non-transcribed genomic regions, leaving the relevance of transcription to the APOBEC mutagenesis in question.

Here, we analyzed the whole genome and transcriptome sequencing data on 505 tumors across 14 cancer types (18), in an attempt to study the connection between the APOBEC mutagenesis and transcription. Our results point on the important role of transcription in APOBEC mutagenesis. That includes higher mutation load in actively expressed genes and on sense strand, presumably driven by the facilitated access of APOBEC enzymes to the single-stranded sense strand during the process of transcription.

## MATERIALS AND METHODS

### Dataset

Somatic alternations in 12 types of human cancer were taken from (18). Indels were filtered out. Five cancer types, BLCA, BRCA, HNSC, LUAD and LUSC, which contained samples enriched with the APOBEC-mutagenesis signature (APOBEC-mutagenesis enrichment > 2.0, calculated as in (8)), were selected for further analysis. Human genome assembly GRCh37/hg19 was used. Calculations were performed in InterSystems IRIS and MATLAB environments. Processing of computation-intensive subtasks was written in C++ and performed on computational cluster.

### Replication timing analysis

Replication timing data for MCF-7, IMR90 and NHEK cell lines were taken from the ENCODE database (19). Replication timing values were divided into seven intervals to create bins containing approximately equal number of the TCN motifs in each bin (Supplementary Tables S1–S3). The mutation density D_APOBEC_ of the APOBEC mutagenesis in the genome regions corresponding to a particular replication timing bin was calculated as the number of single-base substitutions C→T or C→G in the TCN motif divided by the total number of the TCN triplets in these regions: *D*_APOBEC_ = *N*_APOBEC_ / *N*_TCN_. The relative mutation density of the APOBEC mutagenesis was calculated as *RD*_APOBEC_ = *D*_APOBEC_–*D*_NCN_, where *D*_NCN_ is the density of other single-base substitutions in cytosines. The replication data for the IMR90 cell line were used for analysis of the LUAD and LUSC mutational data; the NHEK cell line data, for HNSC and BLCA, and the MCF-7 data, for BRCA. The leading or lagging strand was assigned to the TCN motifs as in (13).

To estimate the statistical significance of the observation that the lagging/leading strand ratio of the APOBEC mutational density is maximal at middle-replicating regions, we repeatedly shuffled mutations between lagging and leading strand for each replication timing bin. For each shuffle, we applied quadratic regression to fit a parabolic curve and to obtain the coefficient of the quadratic term reflecting the curve curvature.

### Gene expression analysis

Gene annotations including gene direction (to infer the sense/antisense strand) were taken from RefSeq (20). Gene-level transcript abundances quantified by RSEM (21) were downloaded from the Broad TCGA GDC (22–26); estimated gene expression levels in the ‘scaled_estimate’ column, representing TPM values according to the description in TCGA wiki, were used. The values of gene expression were divided into seven intervals (Supplementary Table S4). Samples with <600 mutations in genes were excluded. Mutational densities in the expression bins were calculated similarly to the densities in replication timing bins, as described above.

### Mutation clusters and model of mutagenesis

Mutation clusters were defined as described previously (Roberts et al., 2013). Briefly, all groups of at least two mutations in which neighboring changes were separated by 10 kb or less were identified and the *P*-value for each group was calculated under the assumption that all mutations were distributed randomly across the genome as described previously (3). Groups of mutations were identified as clusters if the calculated *P*-value was than 10^−4^ or less. We also introduced additional strict rules for the analysis of mutational clusters—a particular cluster was considered as an APOBEC-induced cluster if all constituent SBS conformed to the APOBEC signature. Similarly, a mutation cluster was considered as non-APOBEC-induced if all cluster’ SBS did not conform to the APOBEC signature. The regression model of the ABOBEC mutagenesis was defined as $NR{D_{APOBEC}}( {r,t} )\ = {\beta _0}\ + {\beta _1}r + {\beta _2}t + \varepsilon$, where *NRD_APOBEC_* is the normalized relative density of APOBEC-induced mutations $NR{D_{APOBEC}}( {r,t} )\ = R{D_{APOBEC}}\ ( {r,t} )/\mathop \sum \limits_{r,t} RD( {r,t} )$. This value is normalized on the sample mutation load to compensate for different time of exposure to mutagens in different samples; $r$ is the replication timing, $t$ is the gene expression level, ${\beta _i}$ are the model coefficients, $\varepsilon$ is the random error.

## RESULTS

### Selection of the APOBEC mutational signature for the analysis of human cancer genomes

To analyze the distribution of APOBEC-induced mutations along the genome and its connection with the replication and transcription one need to distinguish single-base substitutions (SBS) presumably generated by the APOBEC mutagenesis from all other SBS. Such classification of mutational data can be done using the mutational signature attributed to the APOBEC enzymes. Previous studies suggested to use TCW (W stands for A and T) (8) or TCN (7) motifs as the APOBEC mutational signature. Previously, using the TCW mutational signature, we observed the increased density of APOBEC-induced mutations in early-replicating regions (14) supplemented by a small shift in the same direction for the distribution of mutations not conforming to the TCW motif. This observation allowed us to speculate that APOBEC enzymes substantially target DNA outside of the TCW motif in human cancers and to use the TCN motif as more appropriate in this case.

**Figure 1.**
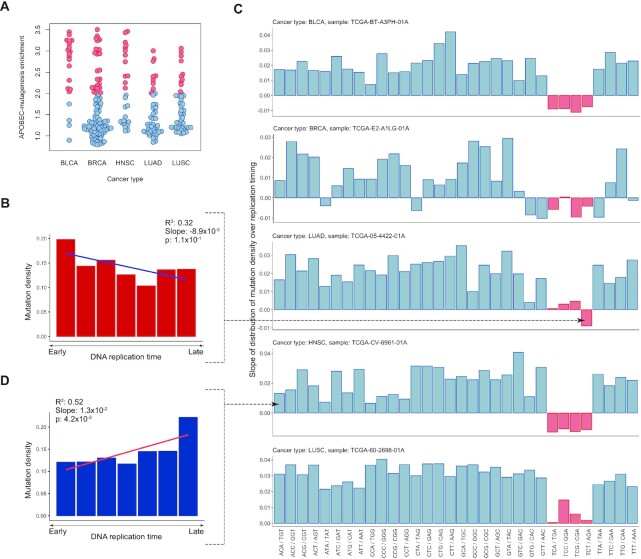
(**A**) Activity of APOBEC mutagenesis in samples from five cancer types. Samples enriched with APOBEC mutagenesis (empirical threshold on enrichment = 2.0) are highlighted in red. (**B** and **C**) Examples of the mutational density distribution over replication timing with positive and negative slopes for the ACA/TGT motif of TCGA-CV-6961–01A sample of the HNSC cancer and the TCT/AGA motif of TCGA-05–4422-01A sample of the LUAD cancer, respectively. (**D**) Representative cancer samples with the calculated slopes of the mutation density distribution over replication timing for all trinucleotide motifs with the substitution in the central nucleotide. Trinucleotides TCN are highlighted in red.

To validate this approach to the considered dataset, we calculated the distributions of the SBS density along the replication timing, while grouping single-base substitutions by their three-nucleotide contexts, i.e. considering all possible 5′ and 3′ bases of the mutated nucleotide (Supplementary File S1). We considered five cancer types having substantial numbers of samples enriched with APOBEC mutagenesis (see Materials and Methods section; Figure 1A), namely, breast carcinoma (BRCA, 96 samples), bladder carcinoma (BLCA, 21 samples), head and neck carcinoma (HNSC, 27 samples), lung adenocarcinoma (LUAD, 46 samples) and lung squamous cell carcinoma (LUSC, 45 samples) (18). As expected, the slope of the density distribution of APOBEC-induced SBS along the replication timing was negative for the TCA and TCT triplets (mutation density decreased toward the late-replicated regions, Figure 1B) and, noticeably, also for the TCC and TCG triplets, as demonstrated for representative samples of five cancer types in Figure 1C. The slopes of the density distribution for all other triplets were mainly positive (Figure 1D). In some cases (Figure 1C, LUSC), slopes of the density distribution for TCN triplets were also positive but still sufficiently smaller than the slopes for other triplets. This apparently reflects the mixed origin of mutagenesis in particular triplets, as each TCN motif contains mixture of mutations generated by different types of mutagenesis, i.e. not only SBSs induced by APOBEC-mutagenesis, but also SBSs generated from other sources. This can offset the effect of higher density of APOBEC-induced mutations in early replication regions, as the mutation density of most cancer signatures is relatively higher in late replication regions (27–33). We argue that similar effects of higher mutation density in early-replicated regions both in TCW and TCS motifs, as well as known TCN specificity of the APOBEC enzymes toward viral DNA, demonstrate that TCC and TCG triplets are also targeted by APOBEC mutagenesis in human cancers, hence supporting our use of a less stringent TCN motif as the APOBEC mutational signature.

### The effect of the high density of APOBEC-induced mutations in early-replicated regions is more prominent in the case of TCN mutational signature

**Figure 2.**
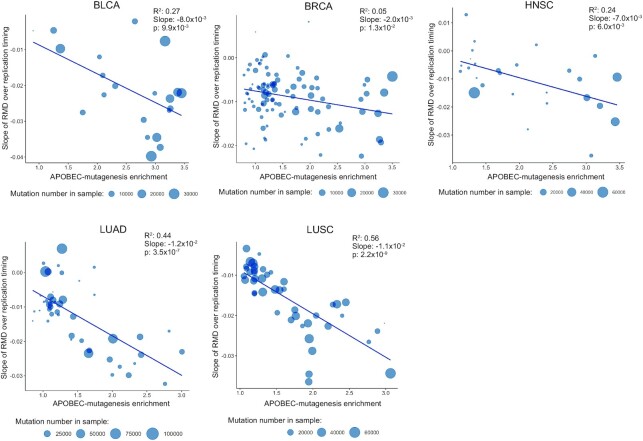
The slopes of the relative mutational density (RMD) distribution (see Materials and Methods section) of APOBEC-induced SBSs (TCN motif) over replication timing as dependent on the activity of APOBEC mutagenesis for samples from five cancer types. The vertical coordinate is the estimated slope of the APOBEC-induced RMD over replication timing as shown in Supplementary File S1 or Figure 1B and C.

Using the newly defined APOBEC mutational signature (TCN motif) we attempt to confirm the earlier observed effect of the increased density of APOBEC-induced SBSs in early-replicated regions in human cancers (14). We calculated the slopes of distributions of the relative APOBEC mutation density over replication timing (see Materials and Methods section). The results for five cancer types are shown in Figure 2. In plots for all cancer types, the slopes of the relative mutation density decrease with the increase of the APOBEC-enrichment of a sample (the APOBEC enrichment is a proxy for the activity of APOBEC enzymes in a particular sample, see Materials and Methods section). This means that the APOBEC mutagenesis rate correlates with the increased density of APOBEC-induced mutations in early-replicating regions. The most profound effects can be seen for BLCA (slope of the regression line, *k* = –8.02 × 10^–3^, *P*-value = 9.92 × 10^–3^), LUAD (*k* = –1.15 × 10^–2^, *P* = 3.44 × 10^–7^) and LUSC (*k* = –1.07 × 10^–2^, *P* = 2.21 × 10^–9^) cancers, moderate effect for HNSC (*k* = –6.61 × 10^–3^, *P* = 6.04 × 10^–3^), and less prominent effect for BRCA (*k* = –2.08 × 10^–3^, *P* = 1.36 × 10^–2^). This effect was not so prominent when only the TCW motif without TCS triplets is used as the APOBEC mutational signature (Supplementary Figure S1) as all slopes of trend lines were higher or equal than those for the TCN motif: BLCA *k* = –4.23 × 10^–3^, *P* = 1.36 × 10^–1^; BRCA *k* = –1.17 × 10^–4^, *P* = 1.36 × 10^–1^; HNSC *k* = –4.49 × 10^–3^, *P* = 4.28 × 10^–2^; LUAD *k* = –1.19 × 10^–2^, *P* = 1.64 × 10^–6^; LUSC *k* = –1.03 × 10^–2^, *P* = 1.19 × 10^–7^.

### APOBEC-mutagenesis is associated with higher mutation density in actively expressed genes

**Figure 3.**
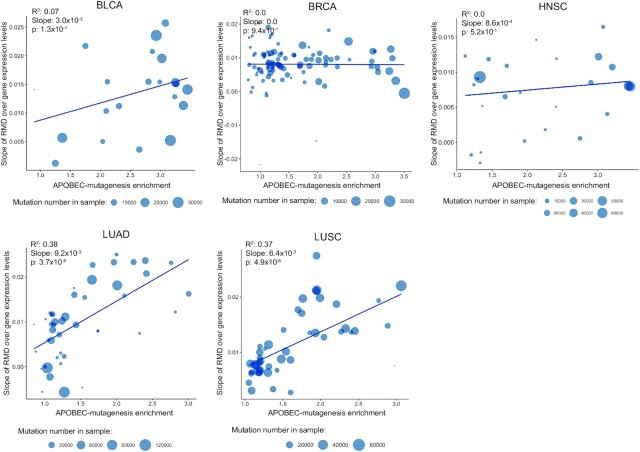
The slopes of the relative mutational density (RMD) distribution of APOBEC-induced SBSs (TCN motif) over gene expression levels as dependent on the activity of APOBEC mutagenesis for samples from five cancer types. The vertical coordinate is the estimated slope of the APOBEC-induced RMD over gene expression levels as shown in Supplementary File S2.

To elucidate possible relationship between transcription and APOBEC mutagenesis, we analyzed gene expression data associated with the studied cancer samples. We estimated the distribution of APOBEC-induced mutations in groups of genes stratified by expression levels. For each cancer sample we divided all genes into seven expression groups (bins) (see Supplementary Table S4) and calculated the mutational density for each bin. Similar to the replication timing analysis, for each bin we calculated the relative APOBEC mutation density as the difference between the density of APOBEC-induced SBSs and other SBSs in cytosines in genome regions associated with the given expression bin. The results for five cancer types are presented in Figure 3. To check that the distribution of mutational density of non-APOBEC-induced SBSs in cytosines is not substantially different from the distributions of SBSs in other nucleotides, we calculated the distributions of mutational density over gene expression levels for all triplets (Supplementary File S2).

Figure 3 shows that stronger APOBEC signature enrichment of the sample corresponds to a steeper slope of the relative mutation density over gene expression levels, i.e. the activity of APOBEC mutagenesis is associated with the increased density of APOBEC-induced mutations specifically in actively expressed genes. This effect is strong for LUAD (slope of the regression line, *k* = 9.22 × 10^–3^, *P*-value = 3.73 × 10^–6^) and LUSC (*k* = 6.4 × 10^–3^, *P* = 4.92 × 10^–6^), weak for BLCA (*k* = 3.0 × 10^–3^, *P* = 1.3 × 10^–1^) and HNSC (*k* = 8.63 × 10^–4^, *P* = 5.24 × 10^–1^), and not visible for BRCA (*k* = –5.96 × 10^–5^, *P* = 9.4 × 10^–1^). This effect is also prominent when the TCW motif is used as the APOBEC mutational signature (Supplementary Figure S2).

### Lagging/leading strand ratio of APOBEC-induced mutational density is maximal at the middle of replicating timing

Further, we investigated how the known effect of high density of APOBEC-induced SBSs on the lagging strand (13,34) relates to the replication timing. Firstly, we confirmed the general effect of increased APOBEC-induced mutational density on the lagging strand by calculating the lagging/leading mutational density ratio for APOBEC-enriched samples (APOBEC enrichment > 2.0) from the considered dataset. We compared the results with ratios calculated for samples with low APOBEC activity (APOBEC enrichment < 2.0). As a control, we considered mutations in cytosines not conforming to the TCN motif in low APOBEC-enriched samples, so as to decrease as much as possible the influence of the APOBEC mutagenesis. Indeed, both low APOBEC-enrichment value of a sample and the mutation triplet not conforming to the APOBEC signature should decrease the probability that mutations taken as a control are APOBEC-induced. The mean lagging/leading mutational density ratio of APOBEC-induced SBSs in APOBEC-enriched samples was 1.35 against 1.0 for SBSs in cytosines excluding the TCN motif of low APOBEC activity samples (Mann–Whitney–Wilcoxon *P*-value = 3.4 × 10^–4^) for BLCA, 1.41 versus 1.05 for BRCA (*P* = 3.3 × 10^–13^), 1.29 versus 1.03 for HNSC (*P* = 1.2 × 10^–7^), 1.24 versus 1.0 for LUAD (*P* = 2.2 × 10^–8^) and 1.3 versus 0.99 for LUSC (*P* = 6.3 × 10^–10^), respectively.

**Figure 4.**
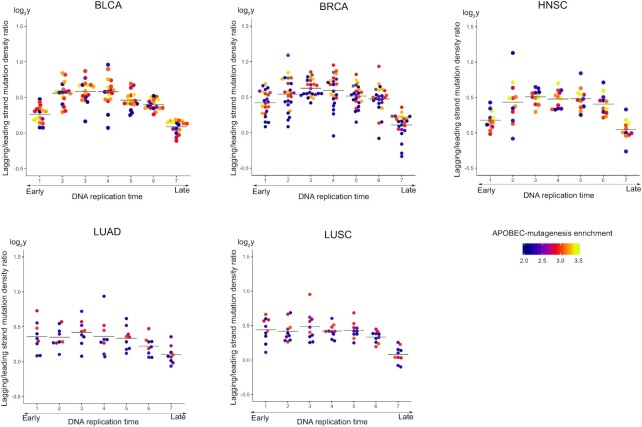
Dependence of the lagging/leading strand ratio of APOBEC-induced SBS density on the replication timing for samples from five cancer types. The horizontal lines show the average lagging/leading strand ratio values.

Then, we measured the lagging/leading strand ratio of APOBEC-induced mutational density along the replication timing (Figure 4). A combination of two known effects, increased density of APOBEC-induced mutations in early-replicating regions and on the lagging strand should yield the highest value of the lagging/leading strand ratio of the APOBEC-induced mutation density in the earliest replication timing bin. However, while in general the lagging/leading strand density ratio decreased from the early to late replication time, surprisingly, the highest values of this ratio were observed in the middle of the replication timing. Thus, the mean value of the lagging/leading strand ratio of APOBEC-induced SBS density over the samples was maximum at the third bin (numbered from early to late replication time) for all types of cancer: BLCA 1.5, BRCA 1.55, HNSC 1.43, LUAD 1.35 and LUSC 1.41. To estimate the statistical significance of this observation, we repeatedly randomly shuffled mutations between the lagging and leading strands (see Materials and Methods section). The calculated *P*-values (BLCA: *P* = 4.8 × 10^–12^, BRCA: *P* = 6.0 × 10^–12^, HNSC: *P* = 1.2 × 10^–8^, LUAD: *P* = 1.8 × 10^–3^, LUSC: *P* = 5.6 × 10^–6^) indicate that the observed effect is statistically significant (Supplementary Figure S10). As a control, we observed that the lagging/leading strand ratio of mutational density over replication timing for other SBSs in cytosines was relatively flat (Supplementary Figure S3). To make sure that the distribution in cytosines not conforming to the APOBEC signature is an appropriate representation of the background mutagenesis, we calculated the distributions of lagging/leading strand mutational density ratio in all triplets (Supplemental File S3) and confirmed it by a manual inspection.

### The prevalence of APOBEC-induced mutations on the sense strand versus the antisense strand positively correlates with the gene expression level

Then, we compared APOBEC mutagenesis between the sense and antisense strands during transcription. We found a statistically significant increase of the APOBEC-induced SBS density on the sense strand, whereas for other SBS in cytosines we observed increased mutational density on the antisense strand. The mean sense/antisense strand density ratio of APOBEC-induced SBSs in APOBEC-enriched samples was 1.13 as compared with 0.74 for SBS in cytosines of low APOBEC activity samples (the Mann–Whitney–Wilcoxon test *P*-value = 6 × 10^–3^) for BLCA, 1.04 versus 0.98 for BRCA (*P* = 8.2 × 10^–2^), 1.03 versus 0.87 for HNSC (*P* = 1.8 × 10^–2^), 1.1 versus 0.65 for LUAD (*P* = 1.3 × 10^–4^), and 1.08 versus 0.69 for LUSC (*P* = 6.3 × 10^–10^), respectively.

**Figure 5.**
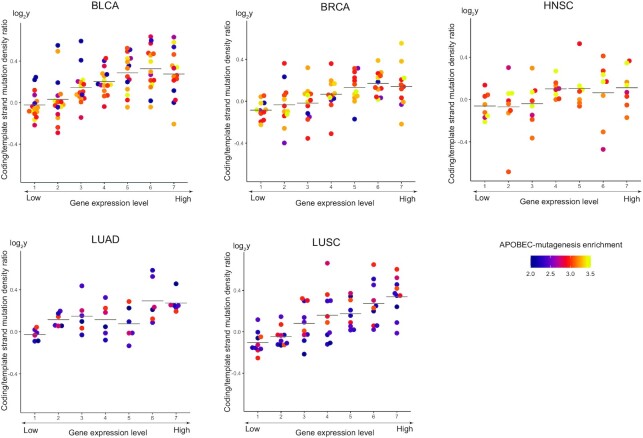
Dependence of the sense/antisense strand ratio of APOBEC-induced SBS density on the gene expression level for samples from five cancer types. The horizontal lines show the average sense/antisense strand values.

We also calculated the sense/antisense strand ratio of the APOBEC-induced mutational density over groups of genes stratified by expression levels. Figure 5 and Supplementary Figure S8a show that the sense/antisense strand ratio of APOBEC-induced SBS density increases with the gene expression level. This effect is observed for all considered cancer types: BLCA (slope of the regression line, *k* = 7.7 × 10^–2^, *P*-value = 1.8 × 10^–13^), BRCA (*k* = 4.7 × 10^–2^, *P* = 1.27 × 10^–6^), HNSC (*k* = 3.65 × 10^–2^, *P* = 2.02 × 10^–2^), LUAD (*k* = 4.21 × 10^–2^, *P* = 4.82 × 10^–4^) and LUSC (*k* = 6.74 × 10^–2^, *P* = 6.63 × 10^–9^). We suggest that this effect is associated both with the availability of the sense strand exposed in the single-stranded conformation for targeting by APOBEC enzymes (35) and with the repairing of the targeted cytosines on the antisense strand by the transcription-coupling repair (TCR) (36–38). Contrary to this tendency, the sense/antisense strand ratio for other SBS in cytosines (Supplementary Figures S4 and S8b, all triplets, Supplementary File S4) decreased with the increasing of gene expression level: BLCA (*k* = –1.5 × 10^–1^, *P* = 7.83 × 10^–3^), BRCA (*k* = –2.47 × 10^–3^, *P* = 8.09 × 10^–3^), HNSC (*k* = –8.66 × 10^–2^, *P* = 5.57 × 10^–3^), LUAD (*k* = –1.37 × 10^–1^, *P* = 1.48 × 10^–12^) and LUSC (*k* = –9.83 × 10^–2^, *P* = 2.74 × 10^–23^). The latter effect might be associated with the smoking-based mutagenesis targeting guanines. Indeed, it is known that in smoking-related tumor genomes, guanine substitutions occur more frequently on the sense strand due to the transcription-coupled repair of the targeted guanines on the antisense strand (39), hence, reducing the density of the SBS in (complementary) cytosines on the sense strand.

We also analyzed whether the density of APOBEC-induced mutations in gene regions depends on the mutual direction of replication and transcription. The comparison of genes with co-direction of replication and transmission and genes with anti-direction of these processes did not yield a statistically significant difference between the dependences of APOBEC-induced mutational density on the gene expression level in these two cases (Supplementary Figure S9).

### Both replication timing and transcription contribute to the mutagenesis by APOBEC enzymes

**Figure 6.**
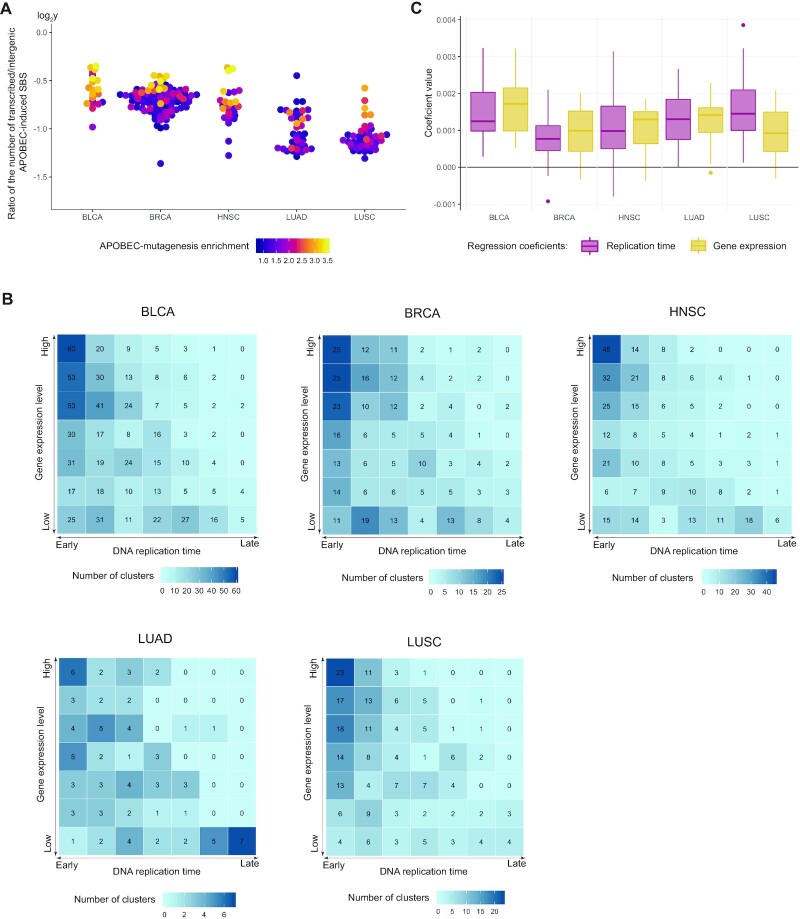
(**A**) Ratio of the number of APOBEC-induced mutations in gene/intergenic regions of samples from five cancer types. (**B**) Distribution of APOBEC-induced mutation clusters over replication timing and gene expression. (**C**) Regression coefficients reflecting the impact of replication timing and gene expression generated by the linear model (see Materials and Methods section) approximating the density of APOBEC-induced SBS in gene regions.

Then, we analyzed whether both replication timing and gene expression influence APOBEC mutagenesis or only one feature is causative and other one is just correlated with the former. Firstly, we calculated the ratios of the transcribed to intergenic number and density of APOBEC-induced SBSs (Figure 6A and Supplementary Figure S5). This showed that when the level of APOBEC mutagenesis increased, the total number and density of APOBEC-induced SBSs in gene regions also increased, in comparison with the total number and density of APOBEC-induced SBSs in intergenic regions. For samples with the strongest APOBEC-enrichment, the total number and density of mutations in transcribed regions increased almost to the level of the corresponding values for intergenic regions. However, as gene-dense regions of the genome is associated with early-replication domains (40), the increase of APOBEC-induced mutations in genes can be associated both with transcription and replication.

To clarify the interdependence of transcription and replication in APOBEC mutagenesis, we calculated the number of SBS clusters over both replication timing and gene expression. Figure 6B shows that, for a particular replication timing bin, the number of APOBEC-induced SBS clusters grows with increasing expression level. The number of APOBEC-induced SBS clusters reaches maximum in regions corresponding to the highest gene expression level of the earliest replication timing bin. Thus, we can conclude that both replication and transcription contribute to the APOBEC mutagenesis. At the opposite, the maximum number of non-APOBEC-induced SBS clusters is concentrated in genome regions corresponding to the lowest expression level of the latest replication bin (Supplementary Figure S6). We also present figures featuring the cluster density instead of the number of clusters by normalizing the numbers of clusters by the sizes of the respective genome regions (Supplementary Figure S7). The observed trends remain the same after cluster normalization.

Then, we estimated the relative impact of the replication timing and gene expression on the APOBEC mutagenesis. For each sample we fit a linear model (see Materials and Methods section) with two independent variables—replication timing and gene expression—and estimated their regression coefficients, reflecting the impact of each genomic feature. The coefficient of the model at the level of gene expression was significant (*P* < 0.05) in 40 out of 53 samples (75%) with very high APOBEC activity (APOBEC enrichment > 2.5) versus 30 out of 76 samples (39%) with very low APOBEC activity (APOBEC enrichment < 1.5). The Pearson correlation coefficient between significance values of the level of gene expression and the replication timing for high and low APOBEC-activity samples was 0.42 versus 0.13, respectively. The coefficients’ absolute values for samples from five cancer types are shown in Figure 6C. As can be seen, the coefficient values of these two features are very close, so it cannot be concluded that one has stronger impact than the other in transcribed regions. We also repeated the same analysis after excluding all clustered mutations. The exclusion of kataegis-like mutations had no substantial impact on the results: the model coefficient at the level of gene expression was significant in 39 out of 53 samples (73%) with very high APOBEC activity versus 30 out of 76 samples (39%) with very low APOBEC activity. The Pearson correlation coefficient between significance values of the replication timing and the level of gene expression for high and low APOBEC-activity samples was 0.4 versus 0.01, respectively.

To validate the obtained results, we also analyzed these data using two additional methods, LMG (41) and analysis of variance (ANOVA). Both methods have confirmed our initial conclusion that the contributions of the replication timing and gene expression both are significant, and their relative impacts are approximately equal (Supplementary Figures S17, S18 and S19), although the results of ANOVA have shown that the relative impact of replication timing was higher in a larger number of samples. Despite the suggested approximately equal impact of replication and transcription, the total number of APOBEC-induced SBSs due to replication, taking into account mutations in intergenic regions, should be considerably larger than the number of SBSs due to transcription.

### Validation on PCAWG dataset

To validate our findings, we repeated the same analysis on a dataset available from the Pan-Cancer Analysis of Whole Genomes (PCAWG) project (42). The PCAWG study is a project for identification of somatic and germline variations in both coding and non-coding regions of >2600 cancer whole genomes across 38 cancer types. Similar to our initial analysis, we have selected cancer types with a substantial number of samples enriched with the APOBEC signature. Six cancer types were selected; five types as in the previous dataset, and the cervical cancer (CESC). It should be noted that most cancer samples of the (18) dataset are also parts of the PCAWG dataset but processed with a different mutation calling procedure. Thus, using the PCAWG dataset, we validated the results on both new cancer samples and types, and on the same samples with an alternative calling procedure. As shown in Supplementary Figures S11–S16, all findings—the increase of APOBEC-induced mutational density in highly expressed genes (Supplementary Figure S12), the peak of elevated density of APOBEC-induced mutations on the lagging strand at the middle of the replication timing (Supplementary Figure S13), the increased APOBEC-induced mutational density on the sense strand (Supplementary Figure S14), and the approximately equal impact of replication and transcription on the APOBEC mutagenesis (Supplementary Figures S15 and S16)—have been confirmed on PCAWG dataset.

## DISCUSSION

While the implication of APOBEC enzymes in human cancer has been discovered 8 years ago (3–5), the mechanisms of APOBEC mutagenesis still are not understood well. The natural suspects are cellular processes associated with temporary unwinding of the DNA into the single-strand state, in particular, replication (13,14) and, possibly, transcription (43,44). Here, we have attempted to disentangle their contribution using whole-genome sequencing and gene expression data for cancers with substantial activity of APOBEC enzymes.

Accounting for the elevated density of APOBEC-induced mutations in early-replicating regions (14), we found indirect but strong evidence that the conventional mutational signature of APOBEC enzymes in human cancers, TCW, can be extended to TCN, as the TCC and TCG triplets also seem to be targeted by APOBEC. Using this mutational signature, we confirmed the higher density of APOBEC-induced mutations in early-replicating regions and found a strong correlation between the density of these mutations in genes and the level of gene expression.

To estimate the relative impact of replication and transcription, we calculated the number of mutation clusters as a function of the replication timing and gene expression, and applied regression analysis to model the number of single-base substitutions. We conclude that both processes influence the activity of APOBEC mutagenesis with approximately equal impact in transcribed regions. The density of APOBEC-induced SBSs is almost equal in intergenic and transcribed regions for samples with the highest activity of APOBEC enzymes, meaning that the fraction of transcriptionally induced APOBEC mutations may be the same as the fraction of transcribed regions in the human genome, that is, about one fourth according to the GENCODE annotation (45).

The results for APOBEC-induced mutational clusters demonstrate stronger effect for bladder, head/neck and breast cancer in comparison with lung cancers. The reason for the stronger effect for lung cancers in isolated (not clustered) mutations may be a better estimate of the background mutagenesis for lung cancers due to the higher number of mutations. As described in the Materials and Methods section, we considered a mixture of APOBEC-induced and background mutagenesis in the TCN motif. It is possible that lung samples with a higher number of mutations allows us to better estimate the level of background mutagenesis and thus to estimate the proportion of APOBEC-induced mutations in more correct way than for samples with lesser number of mutations.

We have also analyzed possible strand asymmetry of APOBEC-induced single-base substitutions both for replication and transcription, and how their density changes over the replication timing and gene expression, respectively. We confirmed higher density of APOBEC-induced mutations on the lagging strand and found an unexpected distribution of the lagging/leading strand ratio of the mutational density over the replication timing, which reaches its maximum at the middle-replicating genome regions. This is not the case for other single-base substitution in cytosines, whose distribution between the replication strands is relatively uniform and independent from the replication timing. We speculate that this effect may be directly linked to the chromatin organization. Indeed, the middle of the replication timing is known for a dramatic switch from replication of euchromatin regions to replication of heterochromatin regions (46).

As for the transcriptional asymmetry, we have observed that the density of APOBEC-induced SBS on the sense strand increases with the gene expression level, while the opposite is observed for other SBS in cytosines. A mechanistic explanation for the former asymmetry might be that the sense strand is exposed during transcription in the single-stranded state via the R-loop formation and hence can be targeted by the APOBEC enzymes, whereas the antisense strand is occupied by the RNA polymerase complex and the RNA–DNA hybrid (Jinks-Robertson and Bhagwat 2014). High rates of transcription are known to promote the R-loop accumulation (47–51). Thus, we speculate that the increased density of APOBEC-induced mutations in highly transcribed genes and on the sense strand can be associated with the increased frequency of the R-loop formation.

The asymmetry observed for other SBS in cytosines could be due to the known smoking-associated damage of guanines and their repair on the antisense strand by transcription-coupled repair. This would lead to the prevalence of guanine substitutions on the sense strand, which is equivalent to the accumulation of cytosines substitutions on the antisense strand. We speculate that in APOBEC-enriched samples this asymmetry is compensated for and switched to the sense-strand cytosine-rich SBS asymmetry due to stronger action of APOBEC enzymes on heavily transcribed genes. Thus, this mutational process, in addition to the transcription-coupled repair of cytosines on the antisense strand, could make the sense-strand cytosine-rich SBS asymmetry associated with the APOBEC mutagenesis stronger than smoking-associated sense-strand guanine-rich SBS asymmetry.

Overall, we have demonstrated an important role of transcription in mutagenesis by APOBEC enzymes in human cancer. Some of our observations, such as the increased density of APOBEC-induced SBS in the sense strand, have simple mechanistic explanations, while others, such as the fact that the lagging strand-associated bias in the density of APOBEC-induced mutations peaks in the middle-replicating regions, remain without underlying molecular mechanisms.

## DATA AVAILABILITY

Source codes are available at https://github.com/intersystems-ru/apobec_expression and https://github.com/mkazanov/molcompbio.

## Supplementary Material

zcab025_Supplemental_File
