# Supplementary material for: Analysis of gene expression and mutation data points on contribution of transcription to the mutagenesis by APOBEC enzymes
Source: NAR Cancer. 2021 Jul 2;3(3):zcab025. doi: 10.1093/narcan/zcab025 (PMC8253550; doi:10.1093/narcan/zcab025)
Supplement: zcab025_Supplemental_File [file zcab025_supplemental_file.pdf]

Supplemental Figures

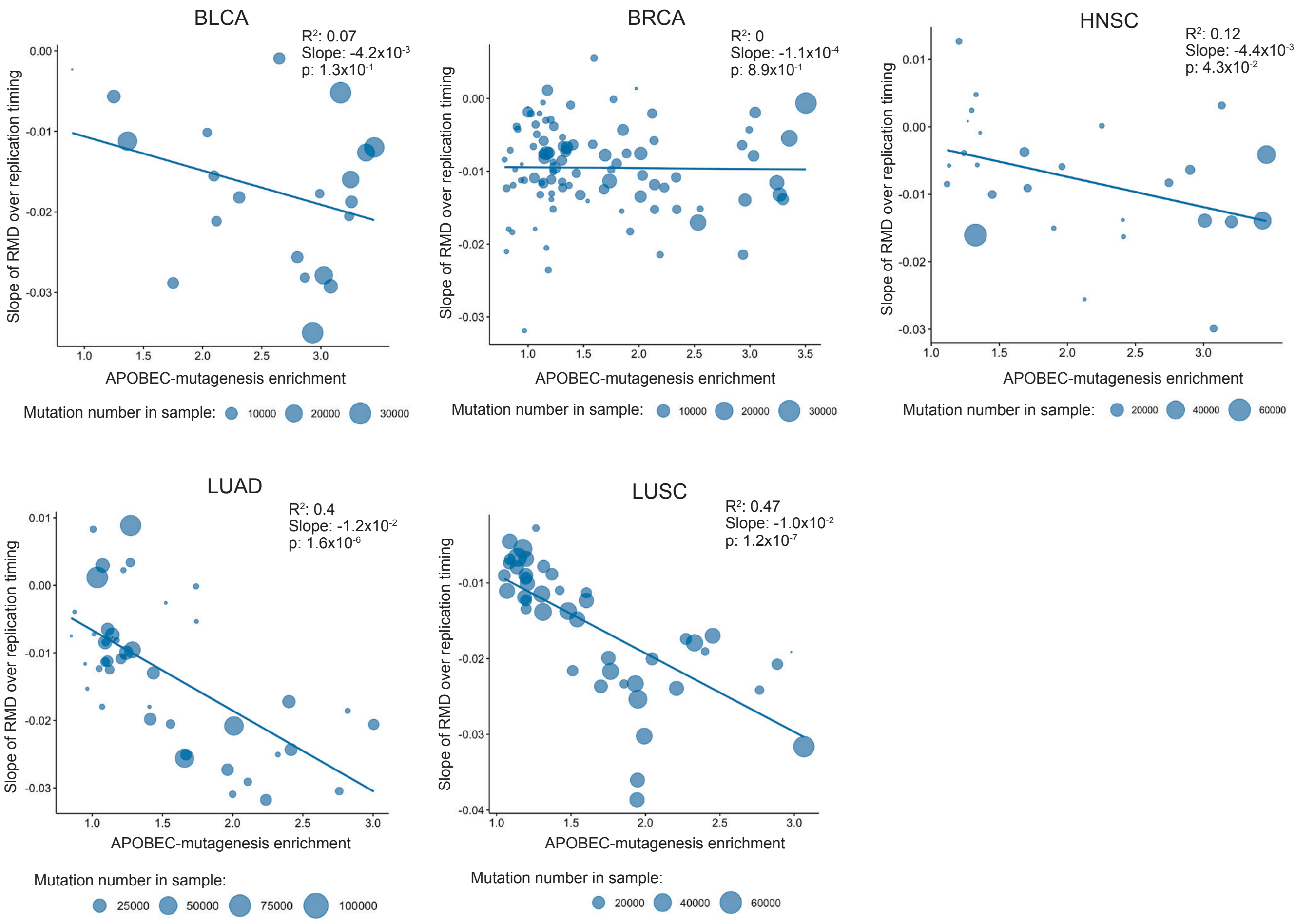

Figure S1. The slopes of the relative mutational density (RMD) distribution (see Methods) of APOBEC-induced SBS (TCW mutational signature) over replication timing as dependent on the activity of APOBEC mutagenesis for samples from five cancer types.

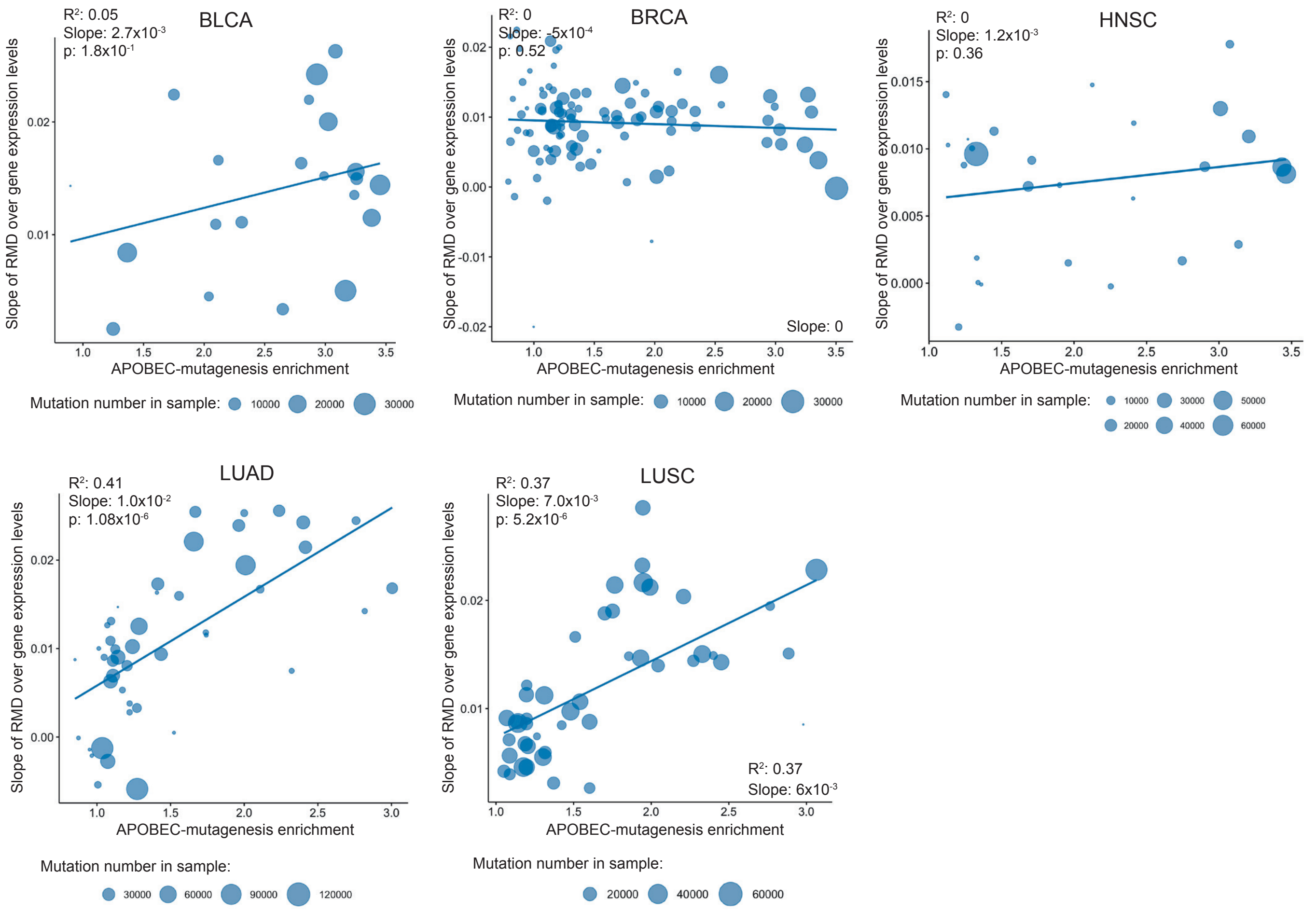

Figure S2. The slopes of the relative mutational density (RMD) distribution of APOBEC-induced SBSs (TCW motif) over gene expression levels as dependent on the activity of APOBEC mutagenesis for samples from five cancer types.

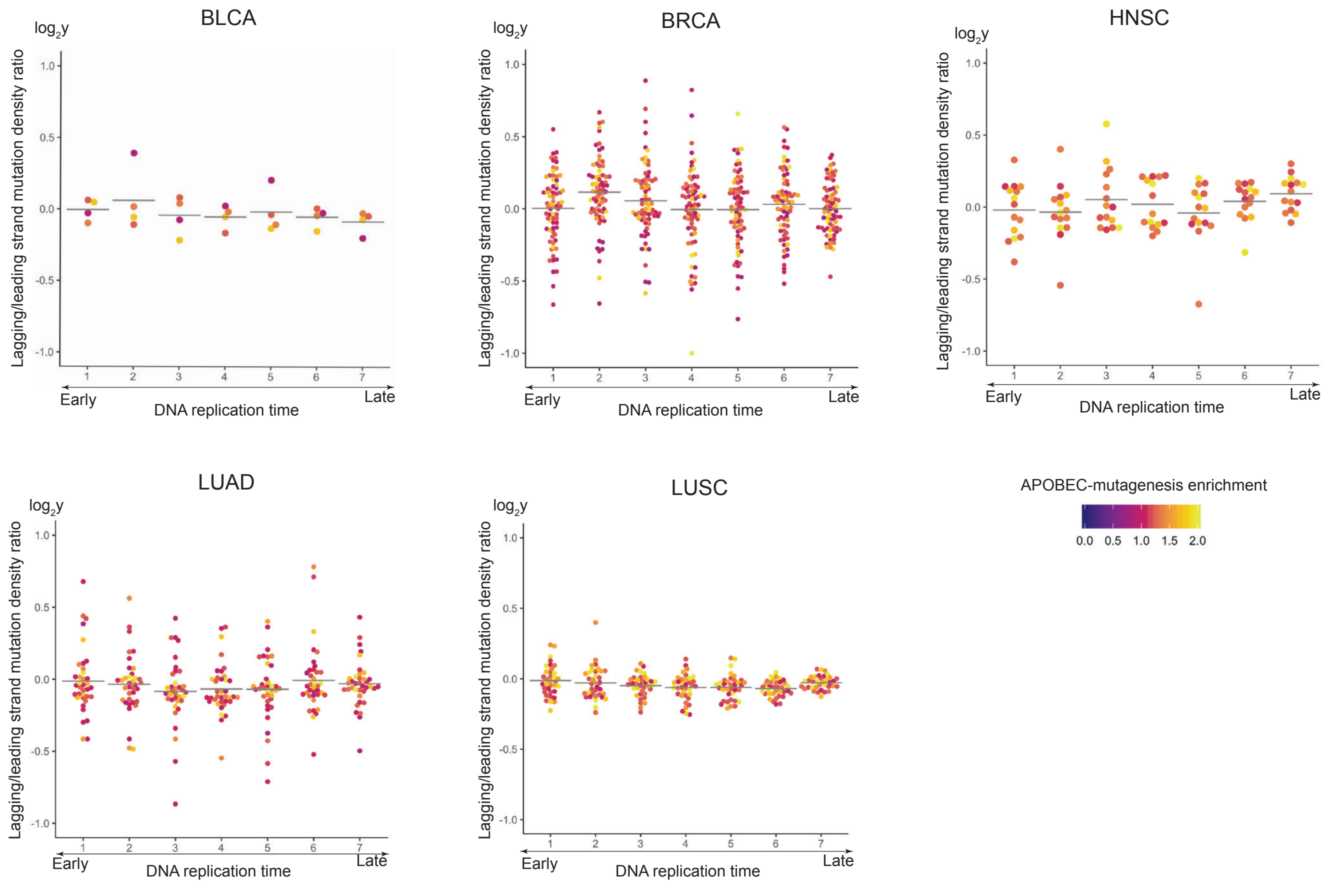

Figure S3. Dependence of the lagging/leading strand ratio of SBS density in cytosines, excluding APOBEC-induced SBS, on the replication timing.

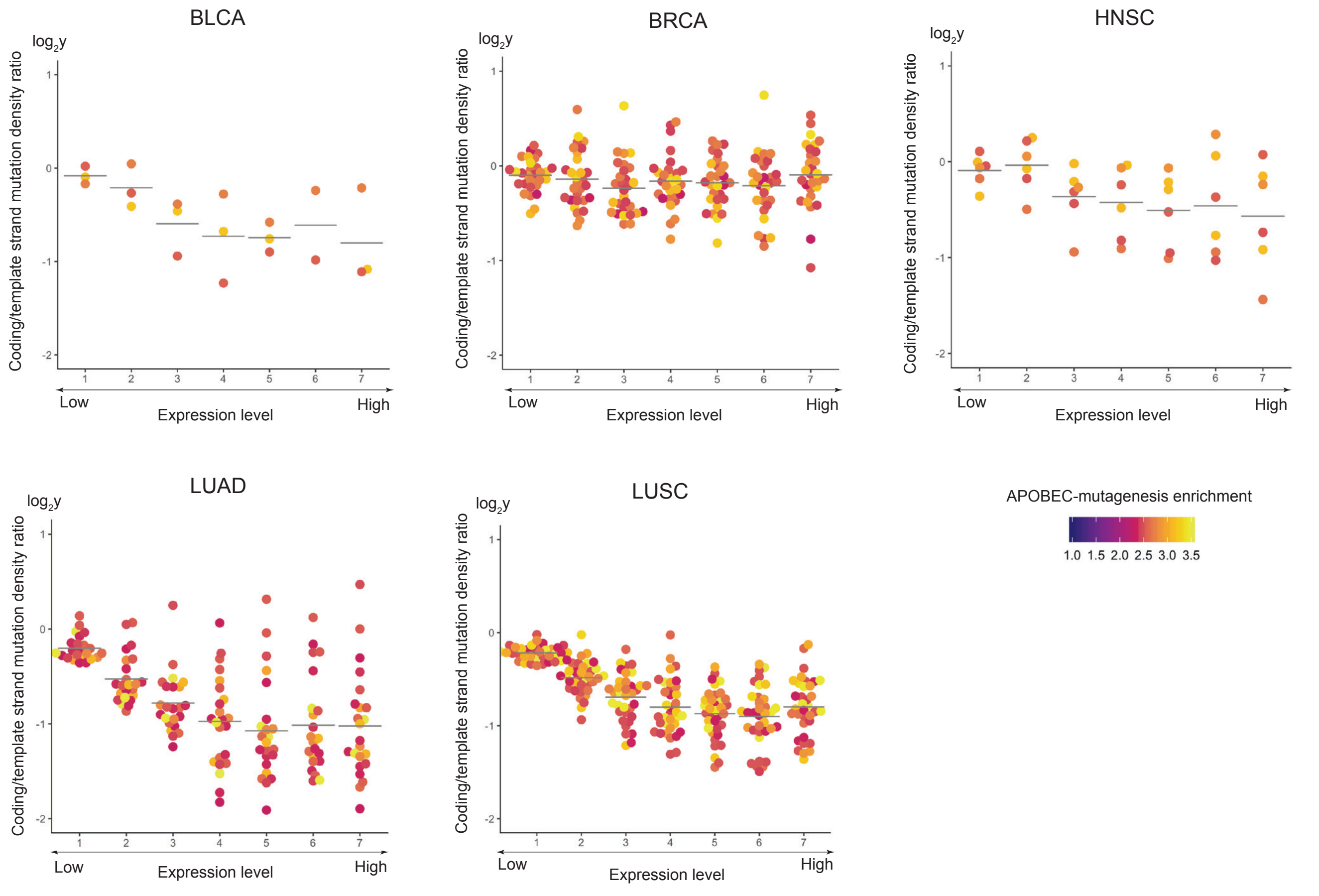

Figure S4. Dependence of the sense/antisense strand ratio of SBS density in cytosines, excluding APOBEC-induced SBS density on the gene expression level.

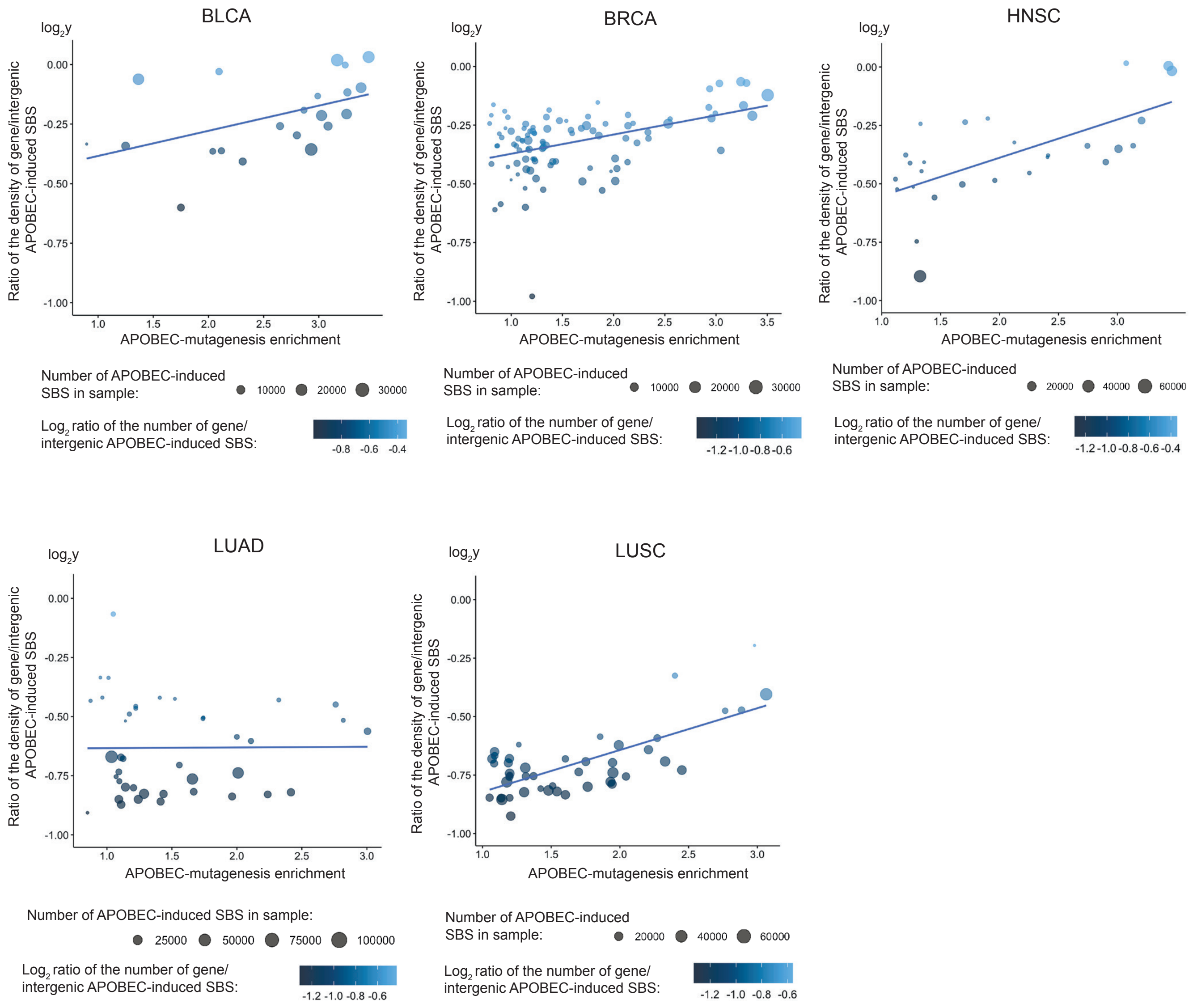

Figure S5. Dependence of the ratio of density and number of APOBEC-induced mutations in gene/intergenic regions on the activity of APOBEC mutagenesis for samples from five cancer types.

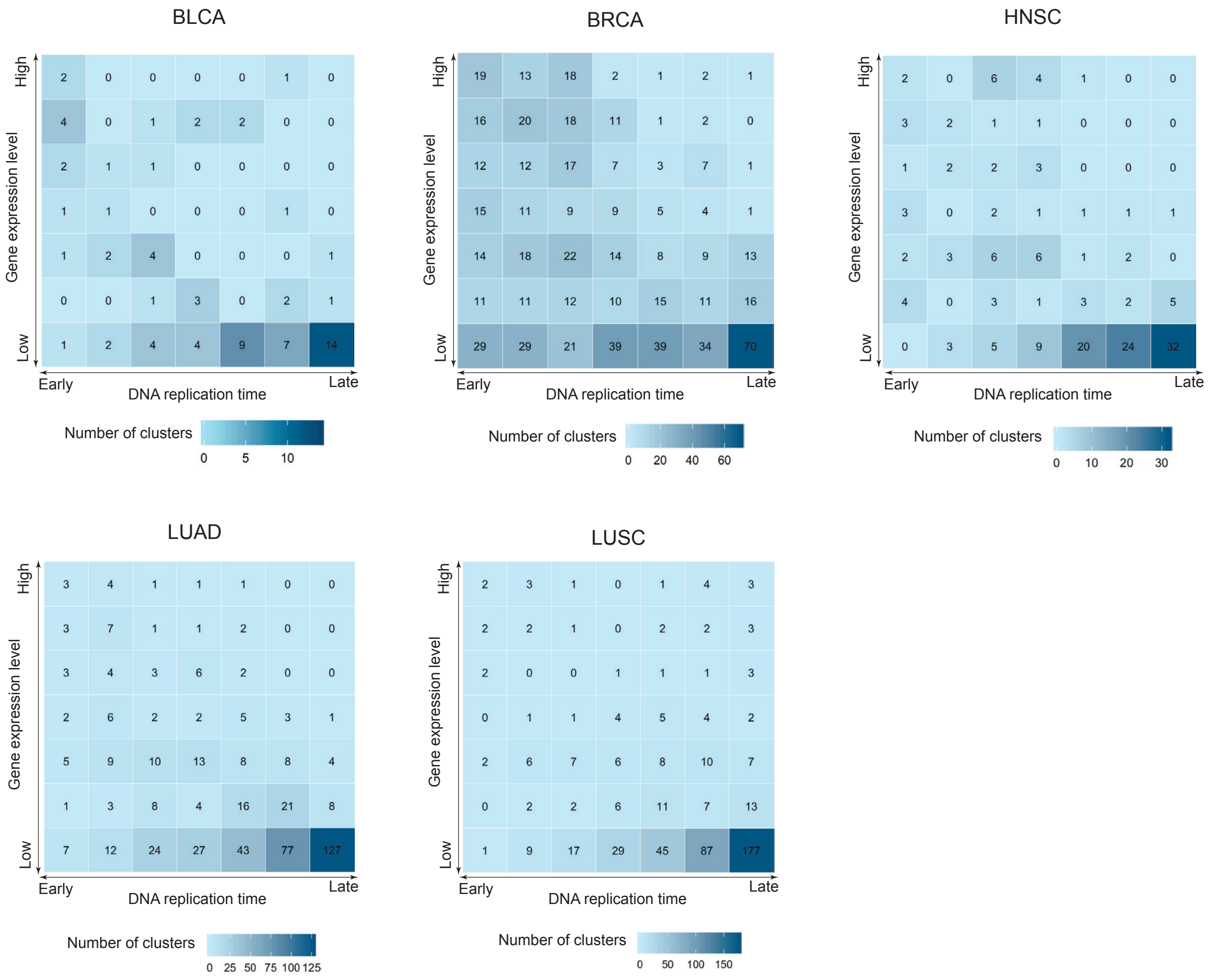

Figure S6. Distribution of non-APOBEC-induced mutation clusters over replication timing and gene expression.

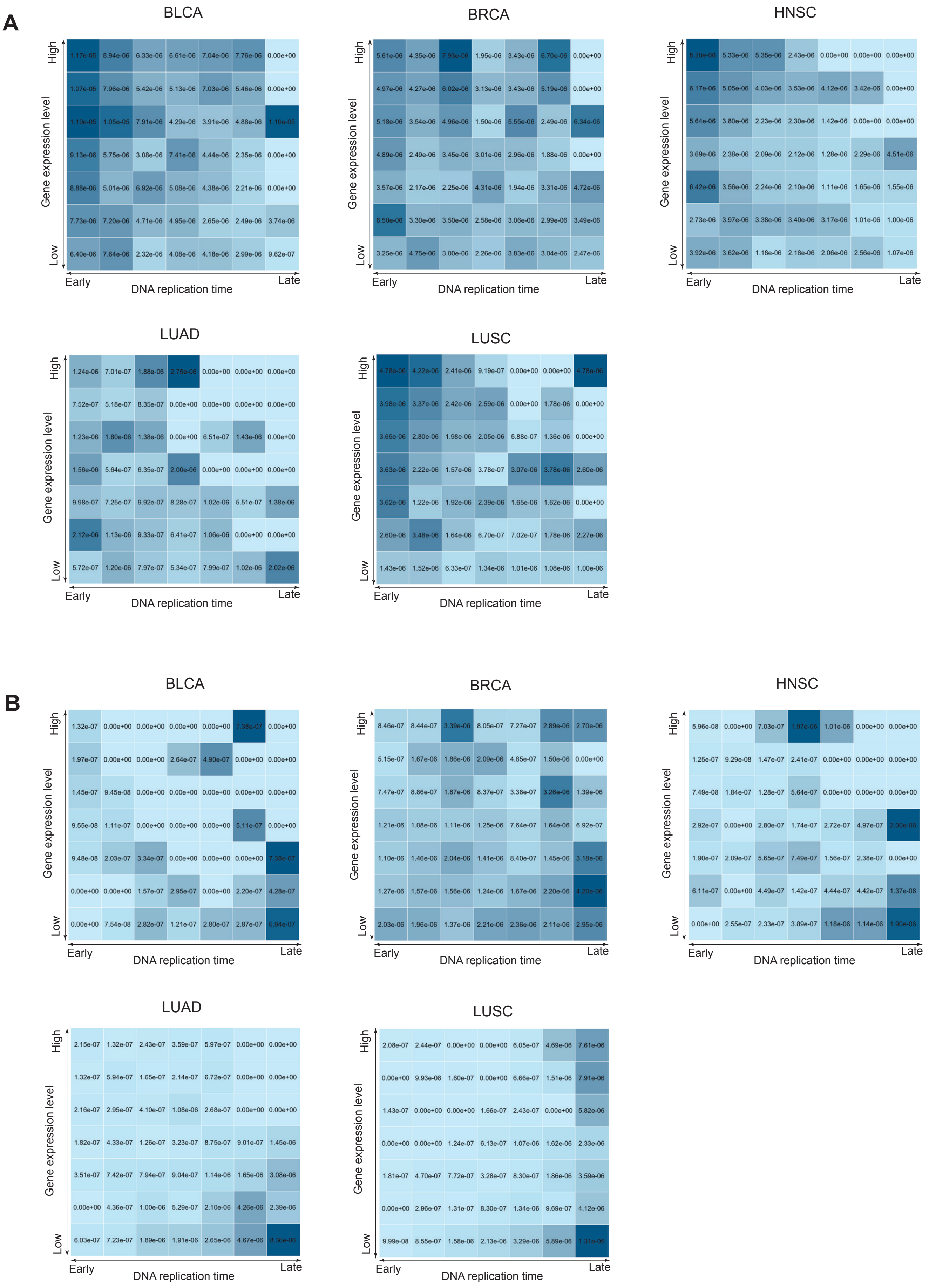

Figure S7. Distribution of the density of (a) APOBEC- and (b) non-APOBEC-induced mutation clusters over replication timing and gene expression.

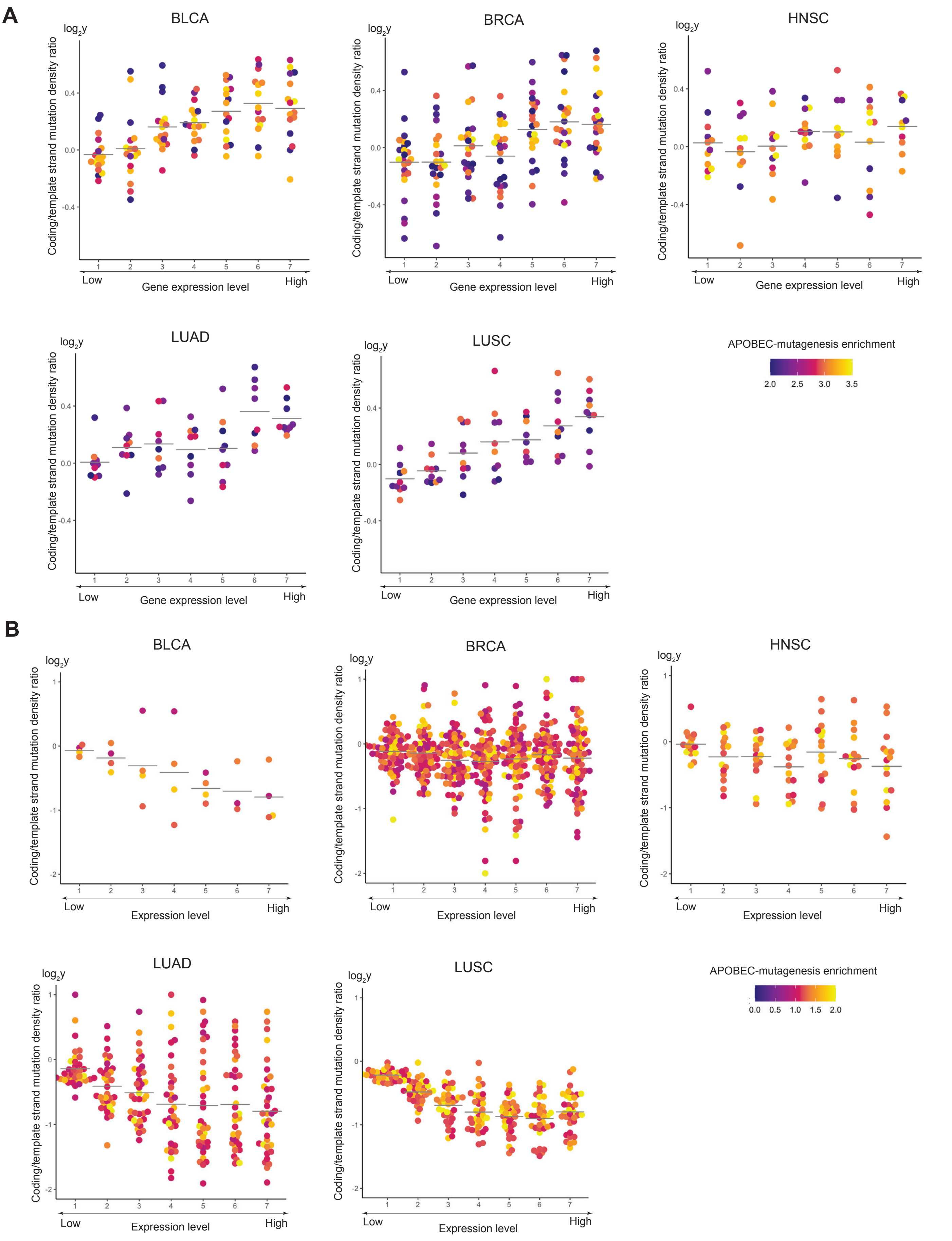

Figure S8. Dependence of the sense/antisense strand ratio of APOBEC-induced SBS density (a) and density of other SBSs in cytosines (b) on the gene expression level. Cancer samples with a low number of mutations and hence higher number of outlier values of sense/antisense strand ratio have not been filtered.

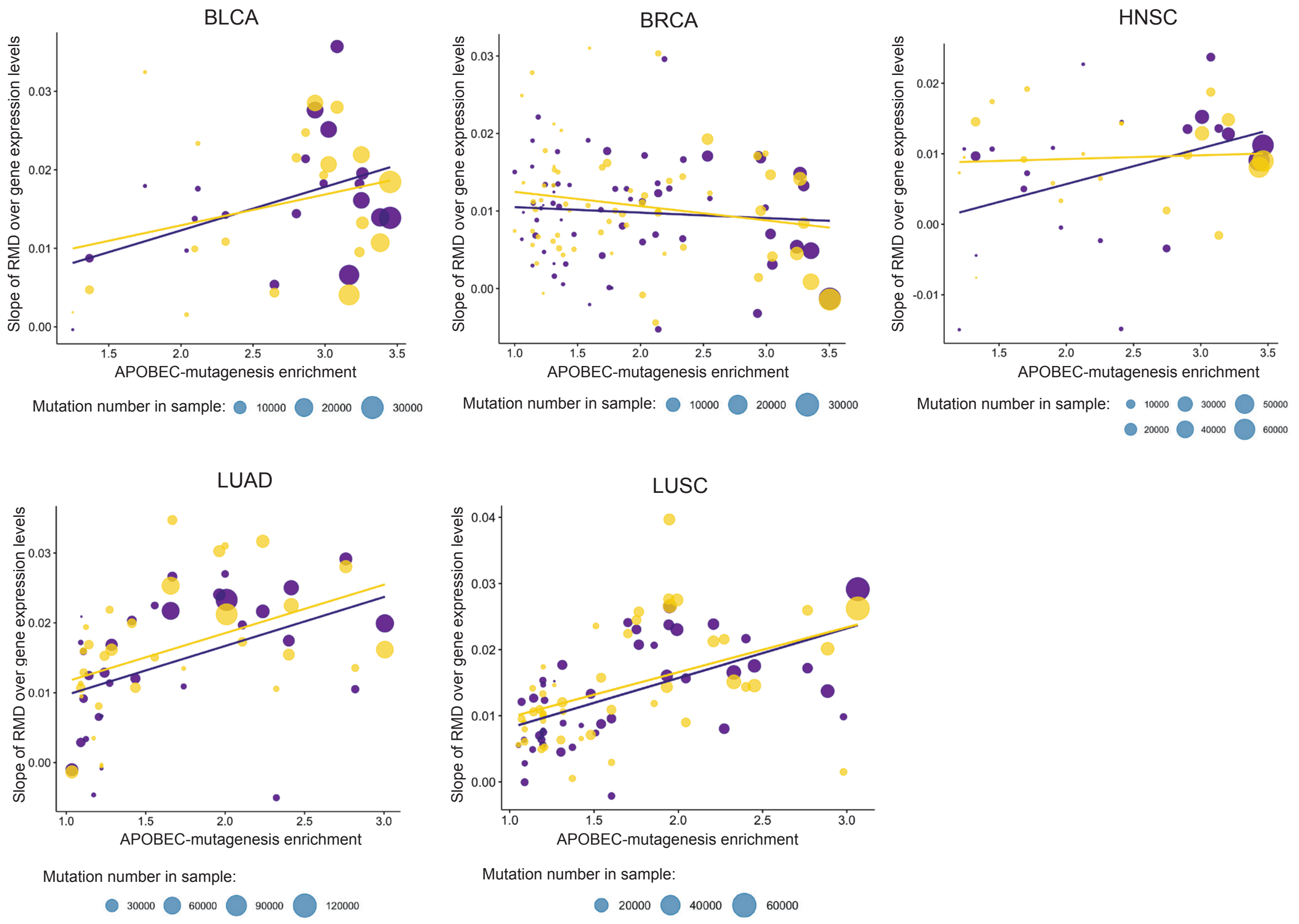

Figure S9. The slopes of the relative mutational density (RMD) distribution of APOBEC-induced SBSs (the TCN motif) over gene expression levels as dependent on the activity of APOBEC mutagenesis for genes transcribed in the direction of replication (violet) and in the opposite direction (yellow).

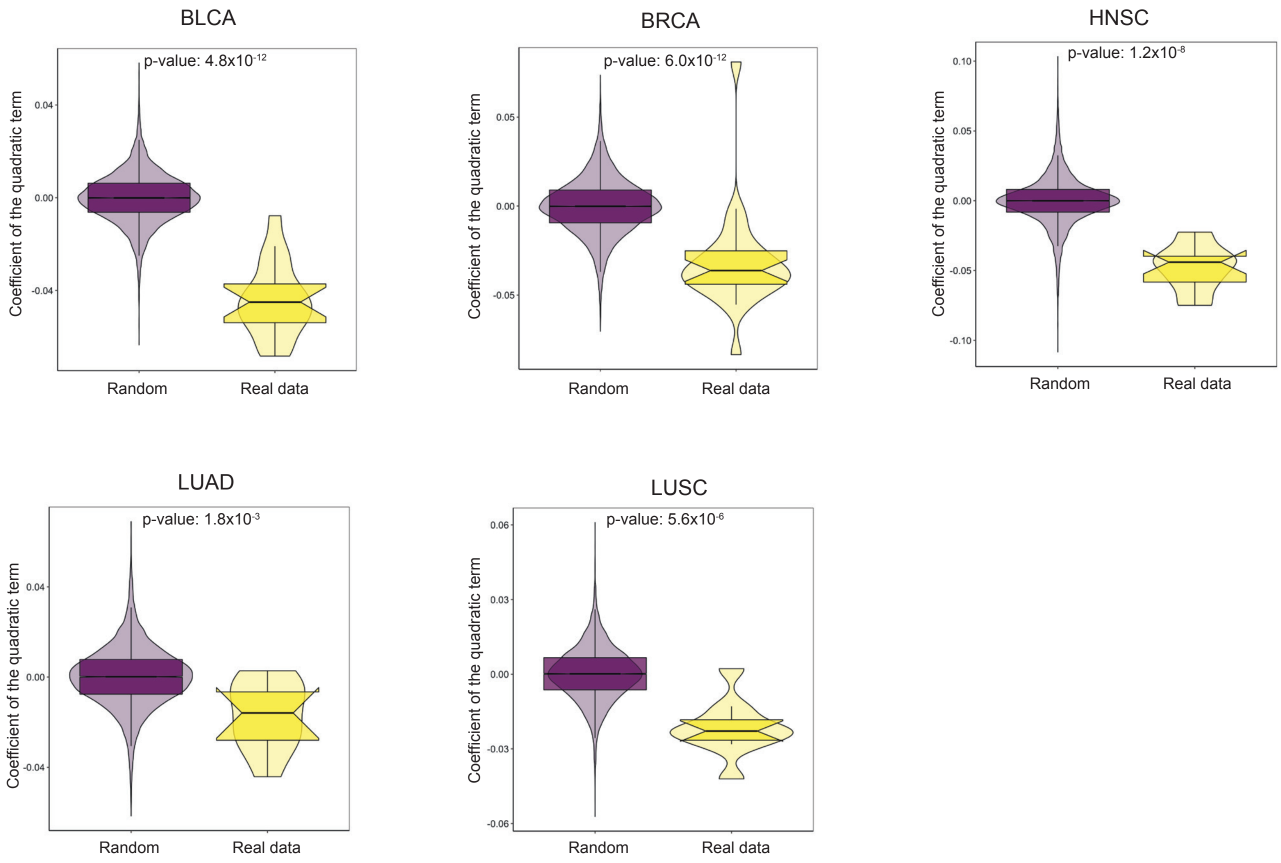

Figure S10. Estimation of the statistical significance for the effect of the highest lagging/leading strand ratio of the APOBEC-induced mutational density at the middle of the replication timing. The distribution of the specified lagging/leading strand ratio along the replication timing was fitted by a quadratic regression and the coefficient of the quadratic term was estimated for each cancer sample. The distribution of the obtained coefficients was compared with the similar distributions obtained from the mutational data where the replication strand was randomly assigned for each mutation.

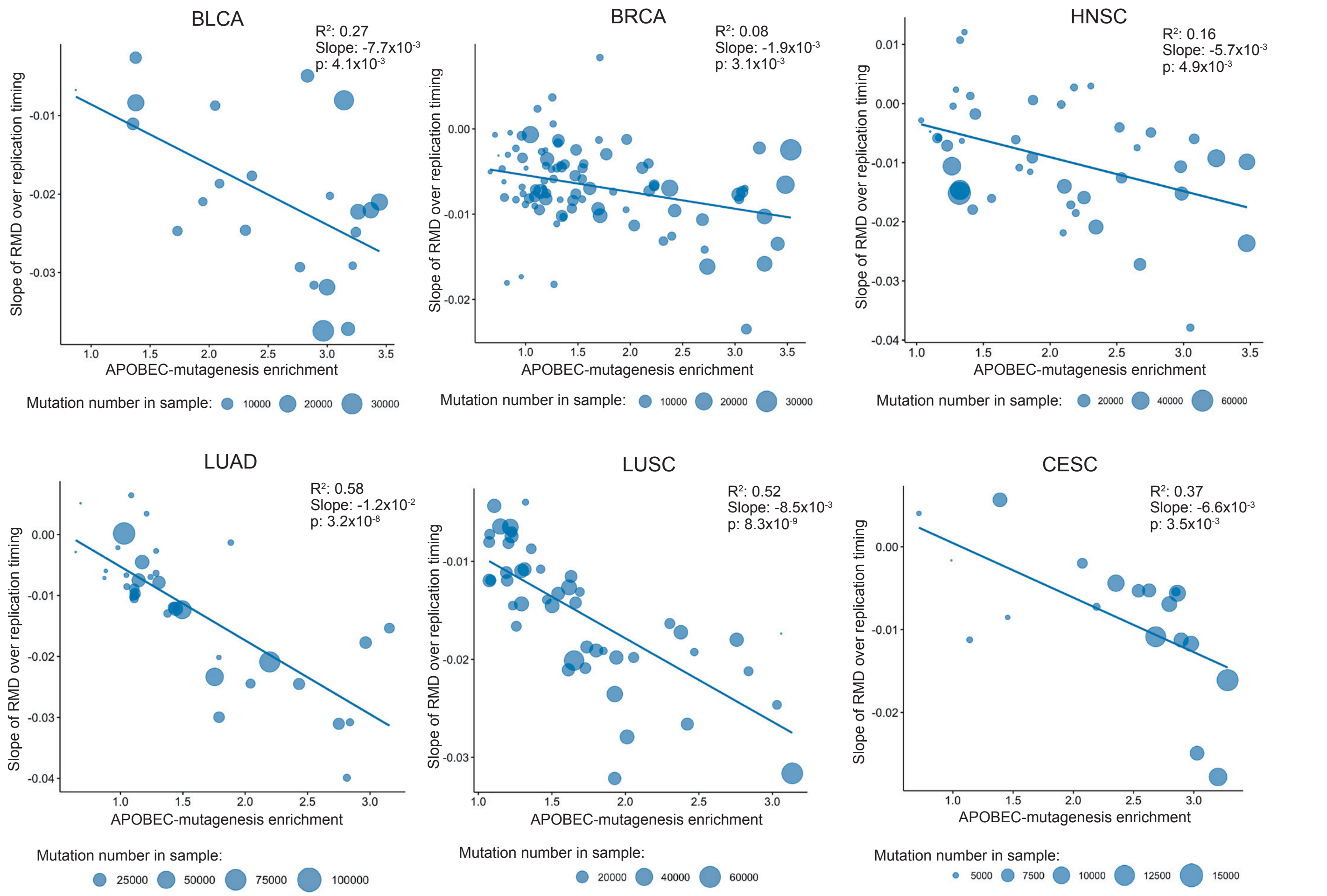

Figure S11. The slopes of the relative mutational density (RMD) distribution of APOBEC-induced SBSs (the TCN motif) over replication timing as dependent on the activity of APOBEC mutagenesis for cancer samples from the PCAWG dataset.

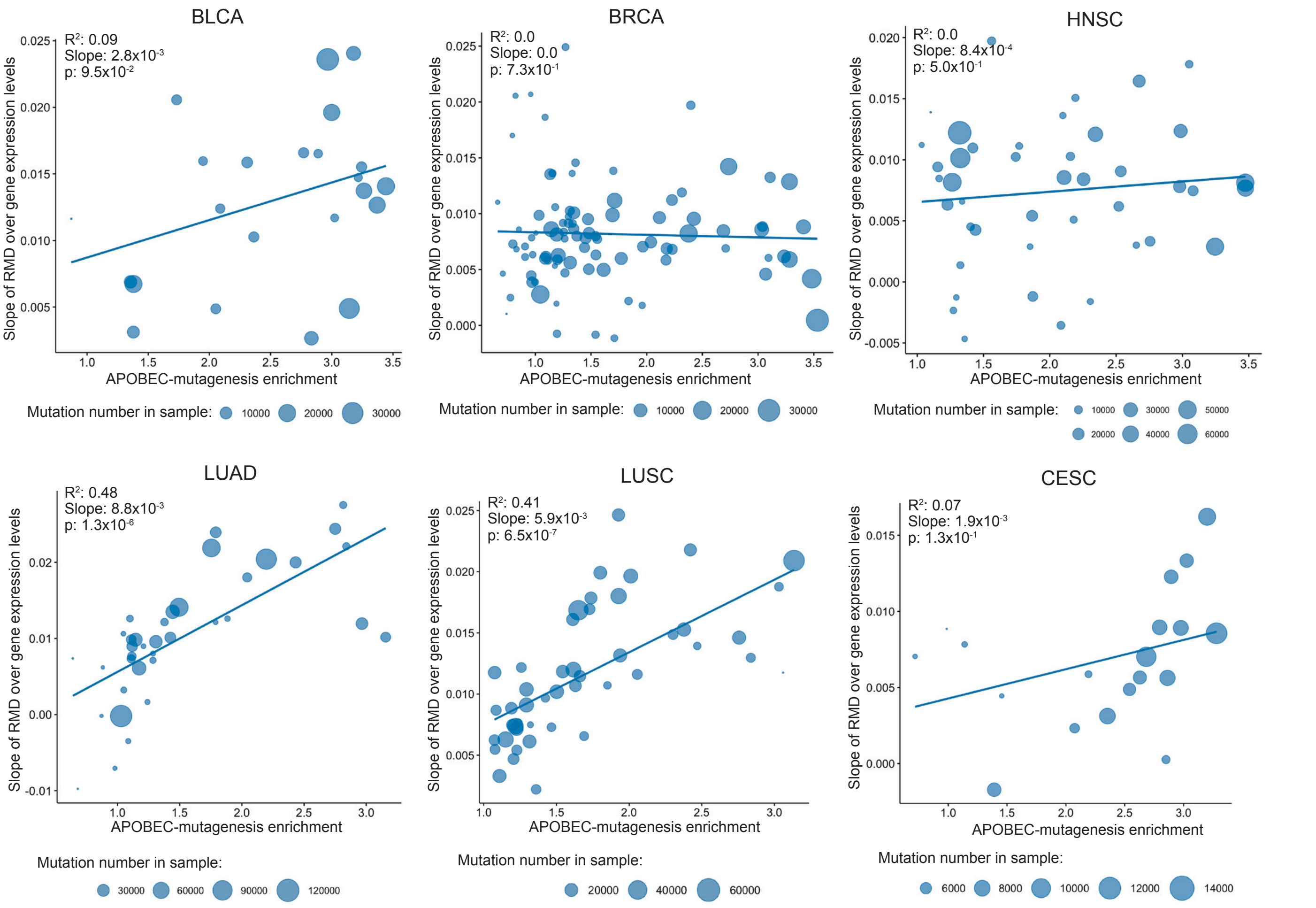

Figure S12. The slopes of the relative mutational density (RMD) distribution of APOBEC-induced SBSs (TCN motif) over gene expression levels as dependent on the activity of APOBEC mutagenesis for cancer samples from the PCAWG dataset.

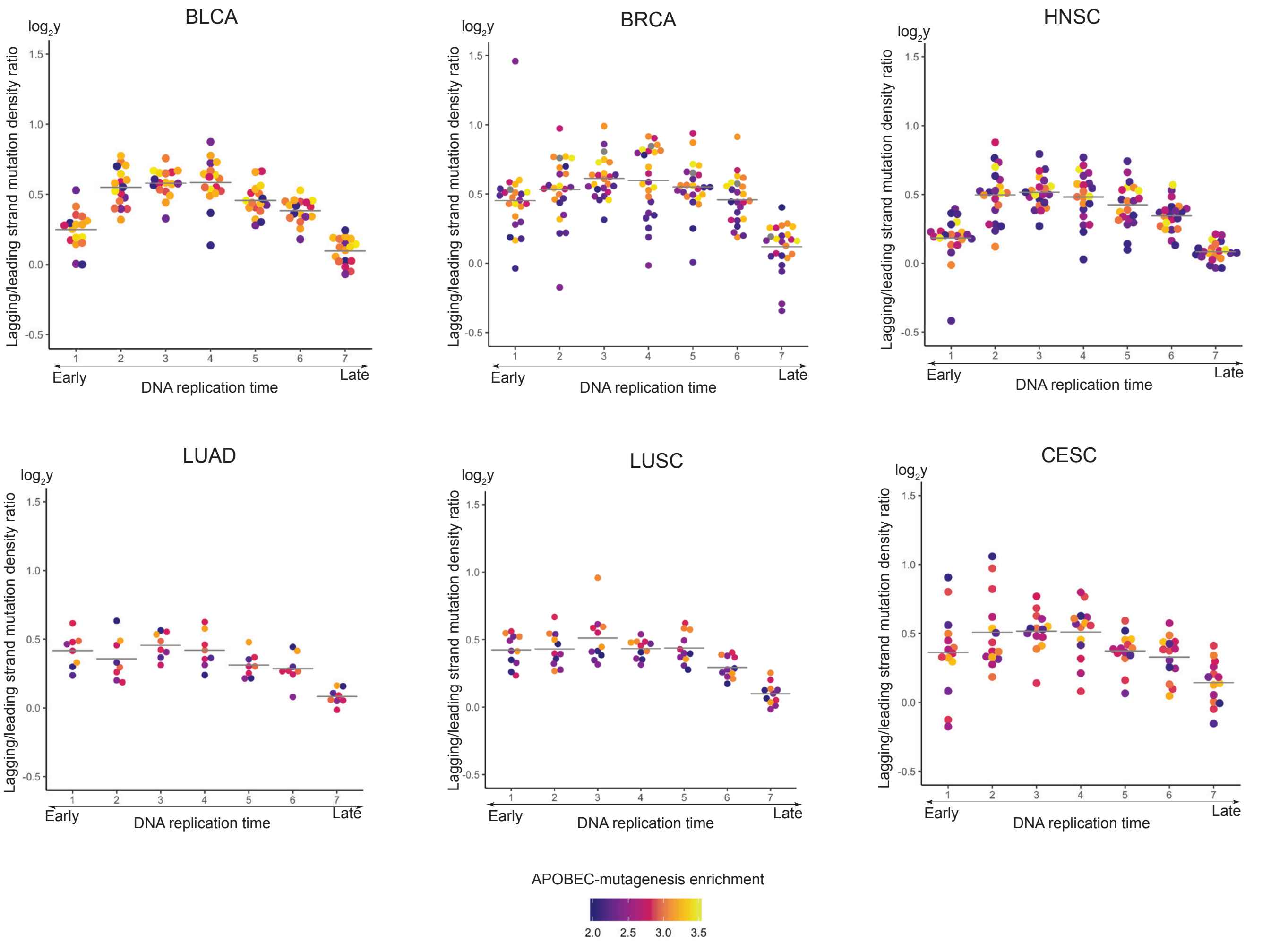

Figure S13. Dependence of the lagging/leading strand ratio of APOBEC-induced SBS density on the replication timing for cancer samples from the PCAWG dataset. The horizontal lines show the mean lagging/leading strand ratio values.

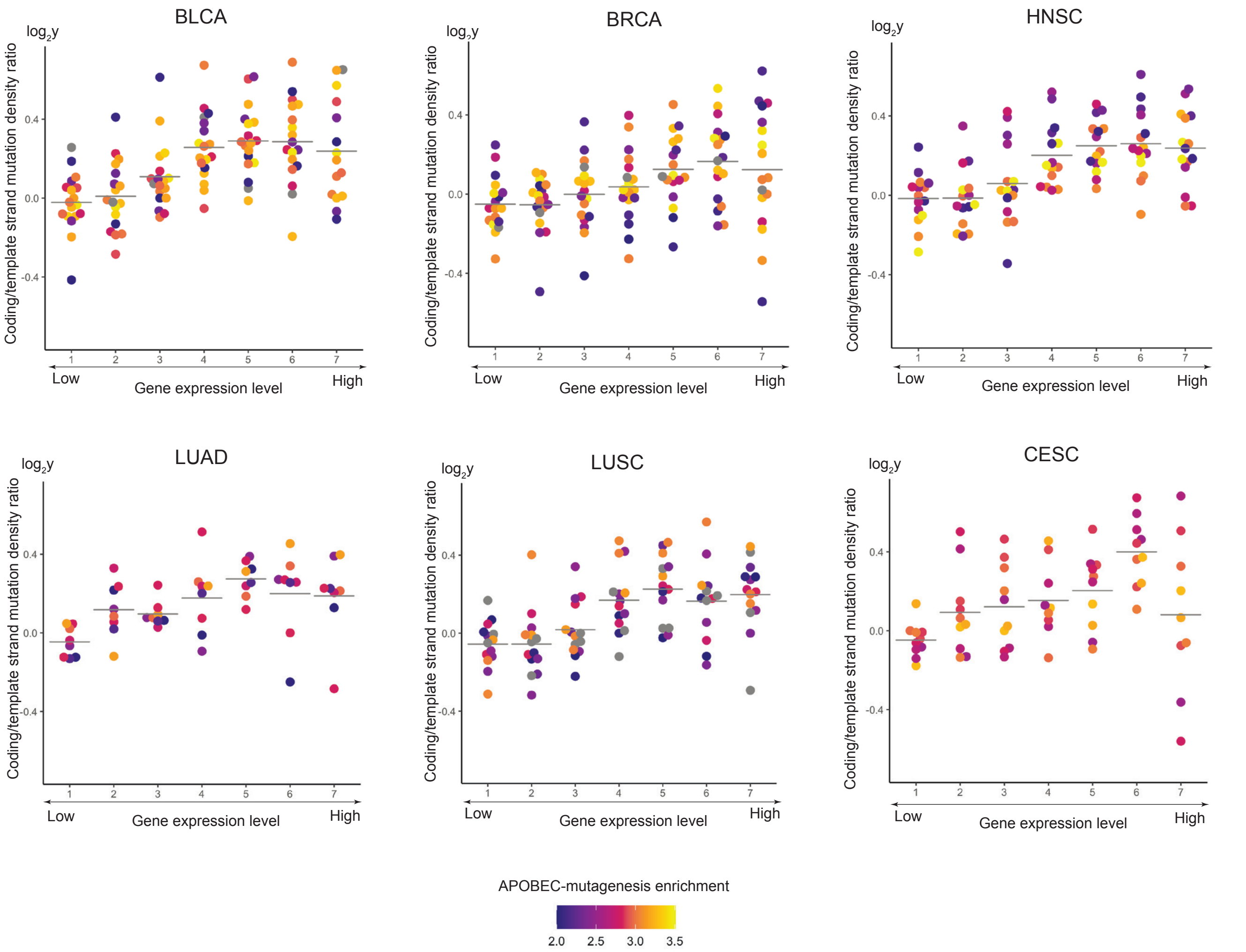

Figure S14. Dependence of the sense/antisense strand ratio of APOBEC-induced SBS density on the gene expression level for cancer samples from the PCAWG dataset. The horizontal lines show the mean sense/antisense strand ratio values.

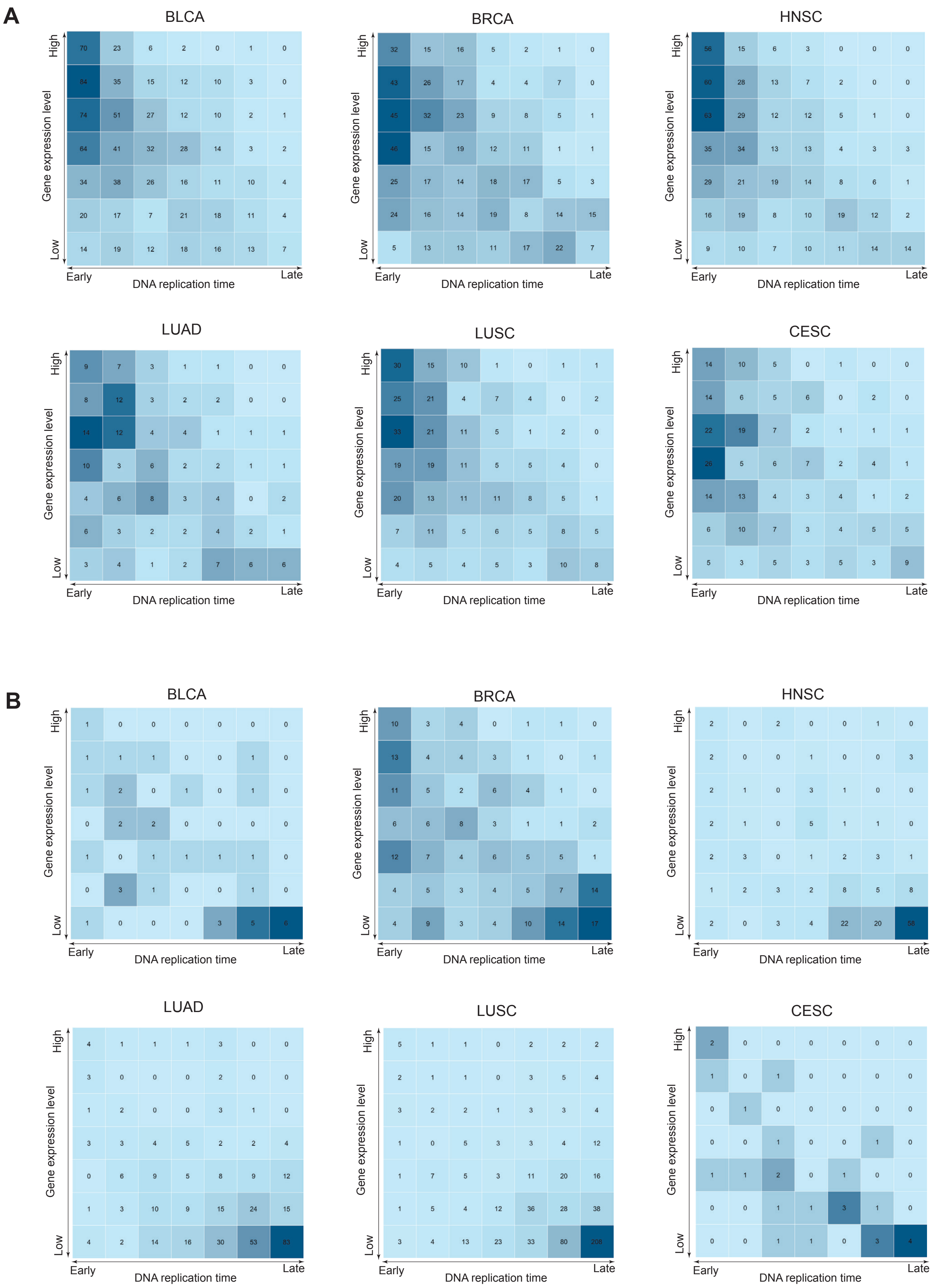

Figure S15. Distribution of the density of (a) APOBEC- and (b) non-APOBEC-induced mutation clusters over replication timing and gene expression for cancer samples from the PCAWG dataset.

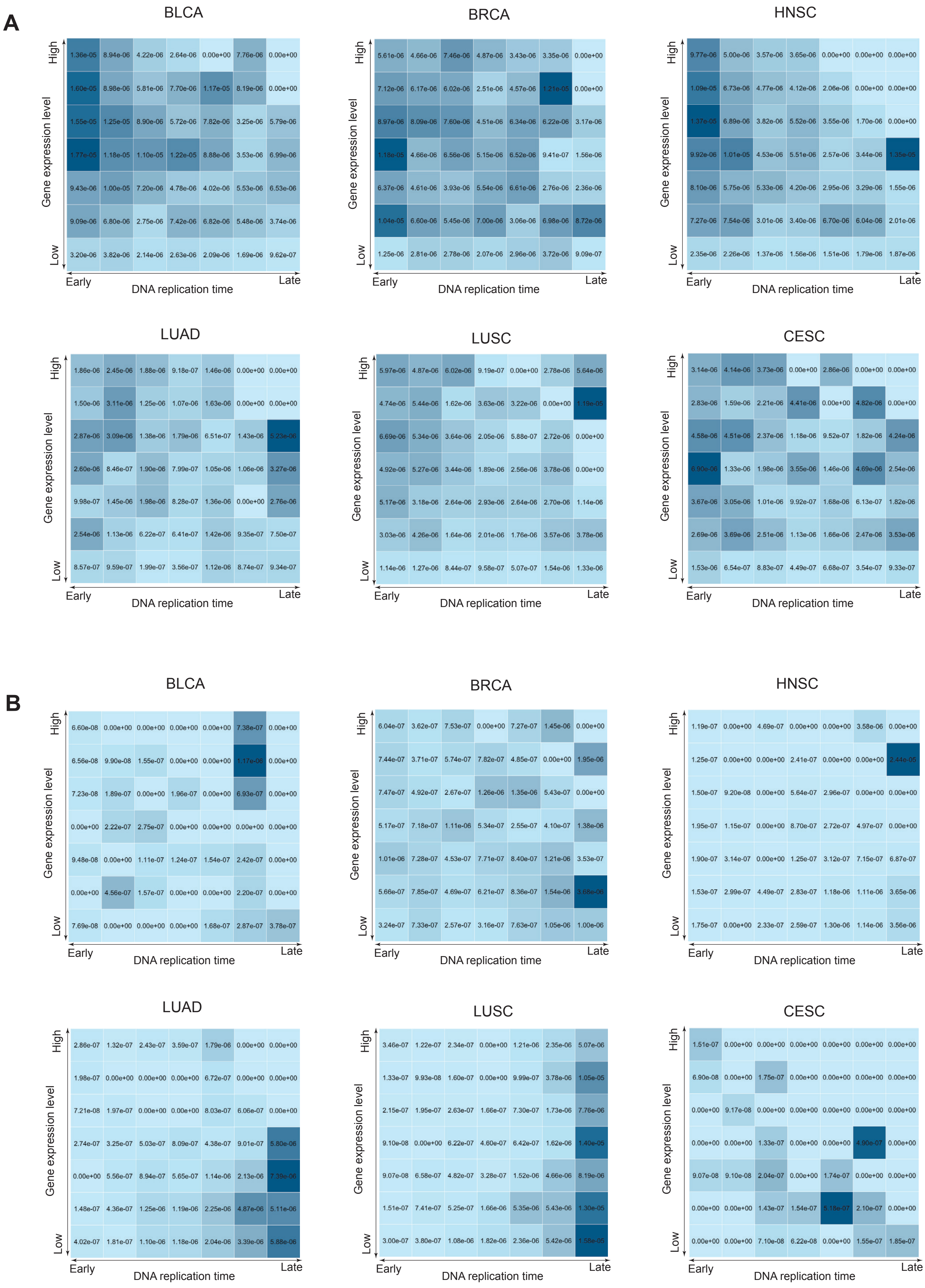

Figure S16. Distribution of the number of (a) APOBEC- and (b) non-APOBEC-induced mutation clusters over replication timing and gene expression for cancer samples from the PCAWG dataset.

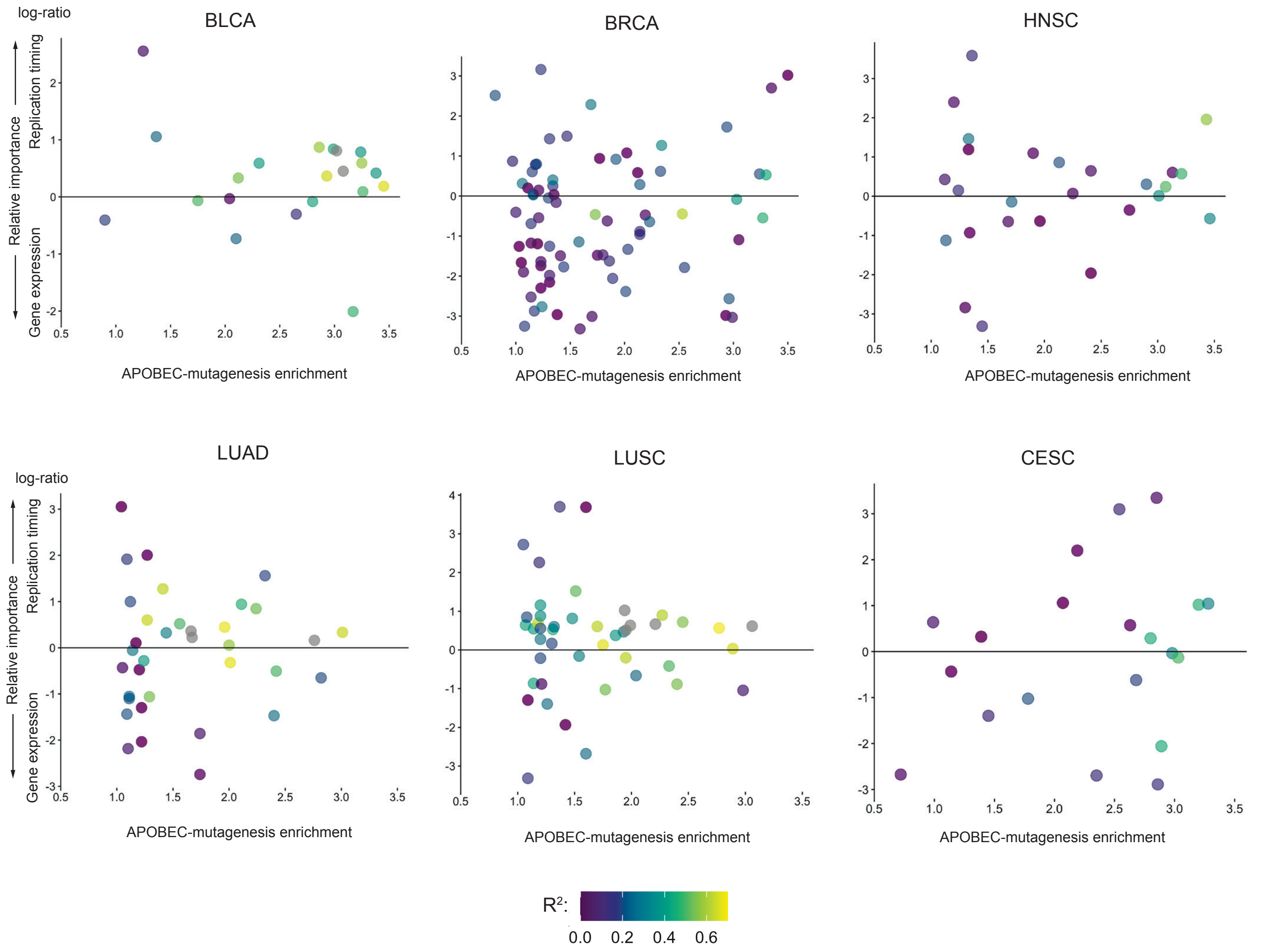

Figure S17. Relative impact of the replication timing and the level of gene expression to the APOBEC-mutagenesis estimated by the LMG method applied to the linear model. The relative importance of the replication timing (I_{RT}) and the level of gene expression (I_{GE}) was estimated as the increase in R^2 averaged over different regressor orderings. The vertical axis represents the logarithm of the ratio of the relative importance of replication timing to the relative importance of gene expression, i.e. $\log(I_{RT}/I_{GE})$.

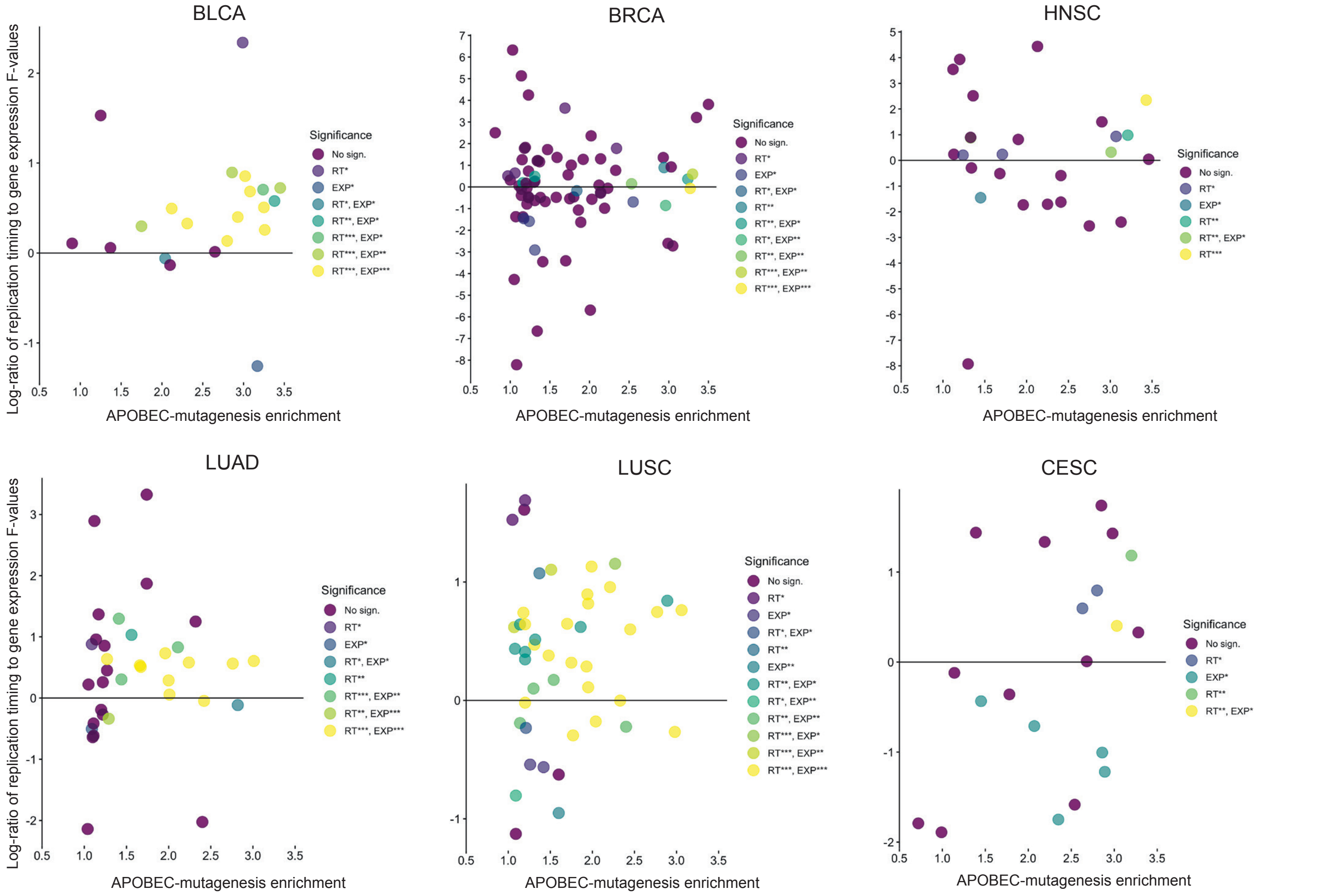

Figure S18. Comparison of the influence of the replication timing and the level of gene expression on the APOBEC mutagenesis, as estimated by the two-way Analysis of Variance (ANOVA). The relative importance of the first (replication timing) and second (level of gene expression) factors is presented as the log-ratio of calculated F-values. Statistical significances of factors are visualized as RT*** ($p<0.001$), RT** ($p<0.01$) and RT* ($p<0.05$) for the replication timing, and as EXP*** ($p<0.001$), EXP** ($p<0.01$), EXP* ($p<0.05$) for the level of gene expression.

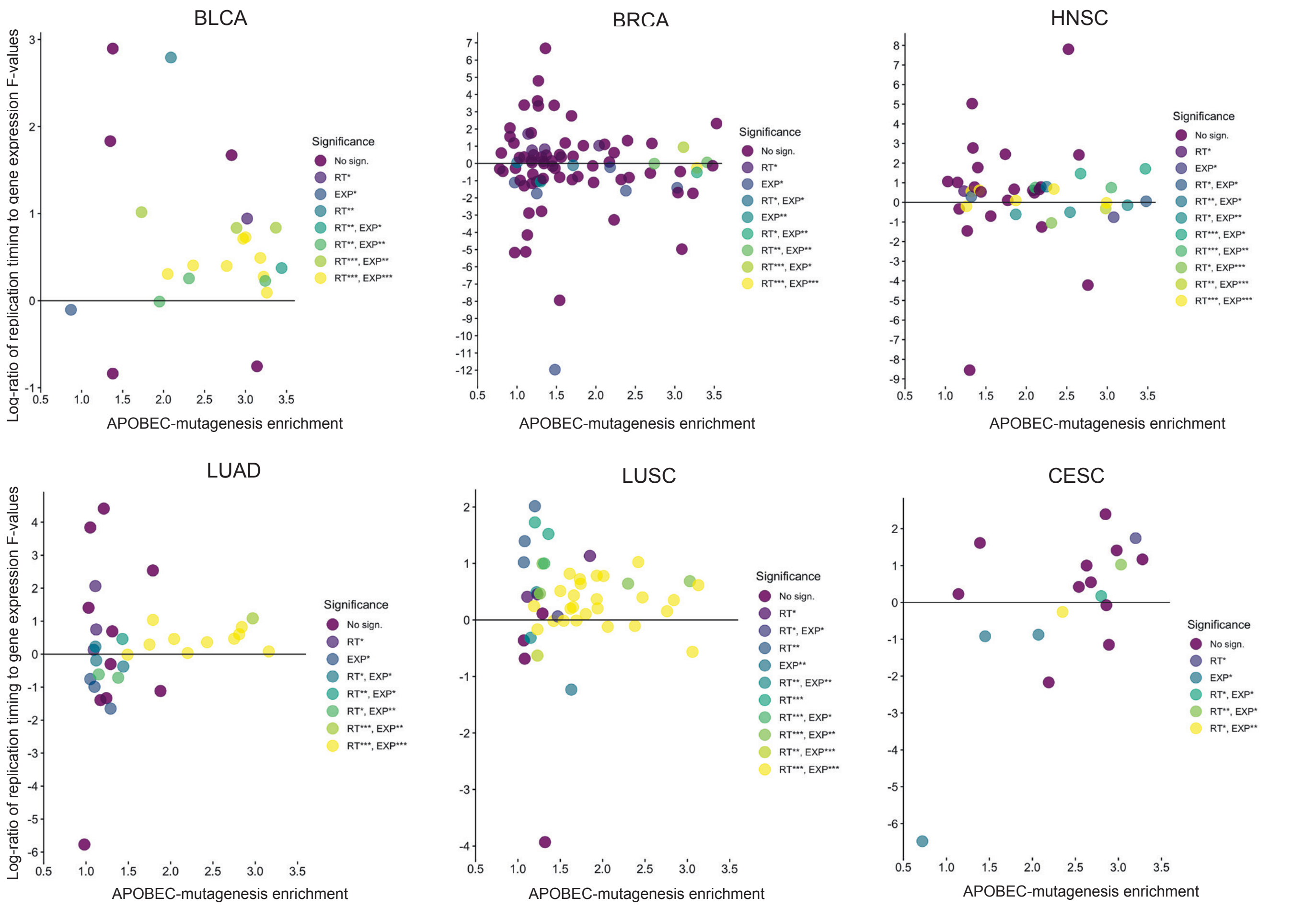

Figure S18. Comparison of the influence of the replication timing and the level of gene expression on the APOBEC mutagenesis, as estimated by the two-way Analysis of Variance (ANOVA) for the mutation dataset after exclusion of positional clusters of mutations (kataegis). The notation is as in Figure S18.

Table S1. Replication timing bins for IMR90 cell

Replication bin	Minimal replication timing value	Maximal replication timing value
1	75	90
2	64	75
3	52	64
4	40	52
5	28	40
6	13	28
7		13

Table S2. Replication timing bins for NHEK cell

Replication bin	Minimal replication timing value	Maximal replication timing value
1	67	80
2	60	67
3	51	60
4	41	51
5	32	41
6	22	32
7		22

Table S3. Replication timing bins for MCF7 cell

Replication bin	Minimal replication timing value	Maximal replication timing value
1	68	86
2	60	68
3	50	60
4	40	50
5	30	40
6	19	30
7		19

Table S4. Gene expression bins

Expression bin	Min expression value	Max expression value
1	0	25
2	25	100
3	100	300
4	300	550
5	550	1000
6	1000	2000
7	2000	

Supplemental File 1:
https://zenodo.org/record/5202856/files/SupplementalFile1.pdf?download=1

Supplemental File 2:
https://zenodo.org/record/5202856/files/SupplementalFile2.pdf?download=1

Supplemental File 3:
https://zenodo.org/record/5202856/files/SupplementalFile3.pdf?download=1

Supplemental File 4:
https://zenodo.org/record/5202856/files/SupplementalFile4.pdf?download=1

.
